# Violation of DNA neighbor exclusion principle in RNA recognition†
†Electronic supplementary information (ESI) available. See DOI: 10.1039/c5sc03740a


**DOI:** 10.1039/c5sc03740a

**Published:** 2016-02-15

**Authors:** Muhammad Yousuf, Il Seung Youn, Jeonghun Yun, Lubna Rasheed, Rosendo Valero, Genggongwo Shi, Kwang S. Kim

**Affiliations:** a Pohang University of Science and Technology , Pohang 790-784 , Korea; b Ulsan National Institute of Science and Technology (UNIST) , Ulsan 44919 , Korea . Email: kimks@unist.ac.kr

## Abstract

DNA intercalation has been very useful for engineering DNA-based functional materials. It is generally expected that the intercalation phenomenon in RNA would be similar to that in DNA. Here we note that the neighbor-exclusion principle is violated in RNA by naphthalene-based cationic probes, in contrast to the fact that it is usually valid in DNA. All the intercalation structures are responsible for the fluorescence, where small naphthalene moieties are intercalated in between bases *via* π–π interactions. The structure is aided by hydrogen bonds between the cationic moieties and the ribose-phosphate backbone, which results in specific selectivity for RNA over DNA. This experimentally observed mechanism is supported by computationally reproducing the fluorescence and CD data. MD simulations confirm the unfolding of RNA due to the intercalation of probes. Elucidation of the mechanism of selective sensing for RNA over DNA would be highly beneficial for dynamical observation of RNA which is essential for studying its biological roles.

## Abstract 2

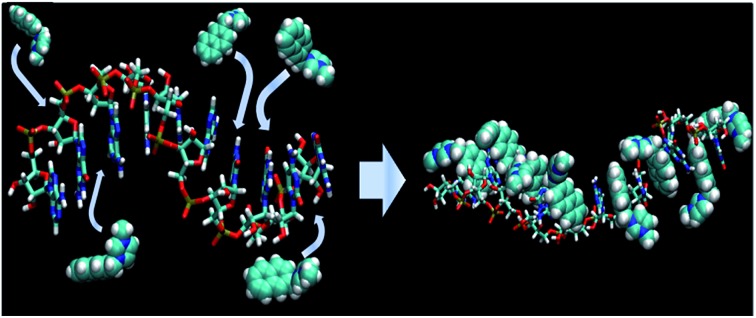
DNA intercalation has been very useful for engineering DNA-based functional materials.

## 1. Introduction

The neighbor-exclusion principle is a well-known rule for intercalative binding of small planar molecules to DNA.^1^–^6^ According to this principle, the two neighboring sites of an occupied intercalation site in DNA must remain unoccupied or, in less absolute terms, intercalation is anti-cooperative at adjacent sites.^3^,^6^ Namely, every second (next-neighbor) intercalation site along the length of the DNA double helix remains unoccupied. The concept of neighbor-exclusion was originally postulated in consideration of possible stereochemical constraints imposed by the sugar-phosphodiester backbone, but the effects of vibrational entropy and counterion release favor the flexible neighbor-exclusion models over the rigid neighbor-exclusion-violating models.^6^ Such neighbor-exclusion states were frequently noted in DNA systems.^1^–^6^ In this study, we show a clear example that such neighbor exclusion states do not work in RNA systems because of differences in the sugar-phosphodiester backbone between DNA and RNA. Specifically, we tested the unique properties of the naphthalene moiety which are responsible for the violation of the neighbor-exclusion principle in RNA, thereby imparting an effect for highly selective recognition of RNA in comparison to DNA.

RNA plays a crucial role as a catalyst inside the ribosome and mediates many transactions in the cell.^7^ In this context, RNA has transformed from a molecule with a minor role in protein synthesis to an important player in molecular biology.^8^,^9^ Thus, the development of RNA detection and recognition technology is gaining immense importance for having an enormous impact on molecular biology and medicine.^10^ The direct visualization of nucleic acids *in vivo* can provide information about the location, kinetics and function of these biomolecules, playing a major role in understanding different inter- and intracellular processes.^11^ Moreover, dynamic quantitative detection of RNA is a vital subject in neurotoxin and cancer biology as variation in RNA abundance is related to gene expression.^12^

Given the diversity of RNA functions, small fluorescent probes that selectively bind to RNA would be a highly efficient approach for therapeutic intervention. Small cationic imaging probes are frequently applied in biological research.^13^–^17^ However, the problem of these small cationic fluorescent probes is that they generally have better affinity for DNA over RNA.^13^–^15^ Chang and co-workers reported two small molecules for RNA detection; however, relatively small differences in affinity were observed between RNA and DNA.^13^–^15^ Yoon and coworkers reported a pyrene based neutral probe which is selective towards RNA compared to DNA but is unselective towards other nucleotides present in biological fluids.^16^ Shirinfar *et al.* reported small naphthalene-imidazolium based cationic cyclophane which can selectively detect RNA over DNA in living cells.^17^ However, the recognition mechanism of the reported probes for RNA was undefined. In this regard, a concise and explicit binding mechanism for fluorescence sensing of RNA is essential for further development of fluorescent molecules showing high selectivity and specificity towards RNA.

**Fig. 1:**
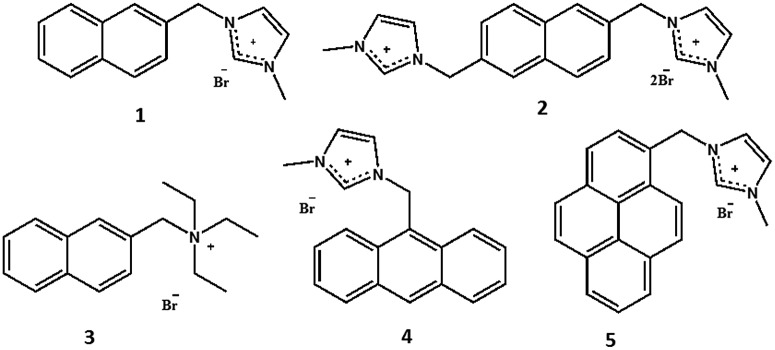
Probes **1–5** (naphthalene based probes **1–3**, anthracene based probe **4** and pyrene based probe **5**).

For the sake of the present study we synthesized naphthalene-, anthracene- and pyrene-based probes (Fig. 1). ^1^H NMR, fluorescence titration, and circular dichroism (CD) experiments were performed to explain the binding stoichiometry and structures. Molecular dynamics (MD) simulations were performed to confirm whether the RNA structure violates the neighbor-exclusion principle within several nanoseconds, which is the time scale for fluorescence. Additionally, density functional theory (DFT) and time-dependent DFT (TD-DFT) methods were used to elucidate the binding and fluorescence mechanisms using stacking nucleobases which exist in the tRNA of baker's yeast: A–A, A–C, A–G, A–U, C–G, C–U, G–G, G–U and U–U pairs (A: adenine; C: cytosine; G: guanine; U: uracil; Fig. S28†). Additional experiments using a probe that replaced the imidazolium moiety with a triethyl amino group clarify the role of charged groups along with the structures suggested by DFT results. Consequently, both the experimental evidence and theoretical calculations indicate the violation of the well-known neighbor-exclusion principle: naphthalene-based small cationic hosts fit into every individual stacking nucleobase with π–π interactions^18^ between the fluorophores and the nucleobases. It is also shown that the entire structure is aided by ionic hydrogen bonding^19^–^21^ between the positively charged moiety and the ribose-phosphate backbone, resulting in selectivity for RNA over DNA. This study on the mode of binding is essential and progressive as no concrete analysis has been reported yet.

## 2. Results and discussion

### 2.1. Fluorescence studies

**Fig. 2:**
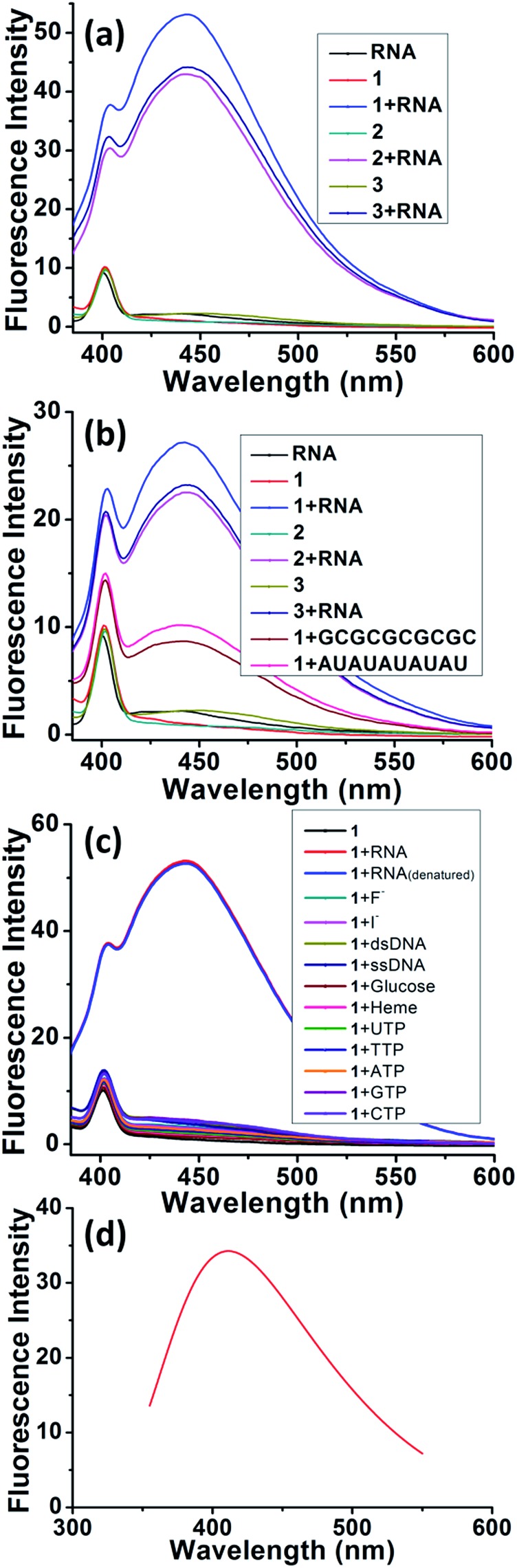
Experimental and theoretically calculated fluorescence spectra. (a) Fluorescence of tRNA from baker's yeast (10 μM), probes **1–3** (10 μM) and probes **1–3** with tRNA from baker's yeast. (HEPES buffer pH = 7.4, slit width = 5 nm) (b) fluorescence of RNA from torula yeast (10 μM), probes **1–3** (10 μM), probes **1–3** with RNA from torula yeast and fluorescence of probe **1** with tRNA (GCGCGCGCGC and AUAUAUAUAU). (HEPES buffer pH = 7.4, slit width = 5 nm) (c) fluorescence of probe **1** (10 μM) in the presence of RNA, denatured RNA, F^–^, Cl^–^, heme, glucose, ssDNA, dsDNA, UTP, TTP, ATP, GTP and CTP (10 equiv.) (HEPES buffer pH = 7.4, slit width = 5 nm). (d) Fluorescence results from TD-DFT calculations.

The details of synthesis of probes **1–5** are described in the ESI (Scheme S1 and S2†).^17^,^22^–^25^ Fluorescence studies have been conducted for selective recognition of RNA because of its simplicity, quick response, and high sensitivity at low concentrations. The fluorescence results of probes **1–5** were checked in aqueous solution at pH 7.4 (10 mM HEPES buffer). The final concentration of tRNA from baker's yeast and RNA from *torula* yeast was determined spectrometrically (*ε*_260_ = 9250 M^–1^ cm^–1^, expressed as molarity of phosphate groups).^26^ Probes **1–3** display lower fluorescence emissions (*λ*_max_ = 402 nm) when irradiated at 350 nm (Fig. 2).^17^ Due to the presence of the quenching effect of the imidazolium moieties in probes **1–2**, negligible fluorescence emission is observed (quantum yield = 0.04 and 0.06 for probes **1** and **2**, respectively) while a quantum yield of 0.05 is observed in the case of probe **3**.^17^,^22^ tRNA from baker's yeast and RNA from *torula* yeast exhibit negligible fluorescence (Fig. 2) but turn-on fluorescence (*λ*_max_ = 443 nm) is observed in the emission spectrum when the probes were treated with tRNA from baker's yeast (quantum yield = 0.63, 0.59, and 0.52 for probes **1–3**, respectively) and RNA from *torula* yeast (quantum yield = 0.41, 0.32 and 0.31 for probes **1–3**, respectively). The turn-on fluorescence (*λ*_max_ = 443 nm) is also observed in the emission spectra for the probe **1** treated with tRNA (GCGCGCGCGC) with a quantum yield = 0.22 and tRNA (AUAUAUAUAU) with a quantum yield = 0.21. This indicates that the fluorescence sensing does not depend on the structural skeleton (Fig. 2). Probe **3** (where the imidazolium group is replaced by a triethyl amino group) gives a similar fluorescence enhancement, indicating that the imidazolium group just gives an electrostatic interaction and has no effect on recognition. Probe **4** exhibits monomer emissions at 398, 421 and 444 nm,^22^ while probe **5** shows monomer emissions at 379, 398 and 419 nm when irradiated at 350 nm.^27^ Both probes **4** and **5** give decreased fluorescence in the monomer when exposed to tRNA, indicating that the naphthalene moiety is responsible for the fluorescence enhancement and hence for the selective recognition of tRNA (Fig. S11†). The new peak at ∼443 nm upon binding of probes **1–3** with tRNA from baker's yeast, RNA from *torula* yeast, tRNA (GCGCGCGCGC) and tRNA (AUAUAUAUAU) is attributed to excimer formation.^17^ The absorption spectra of probes **1–3** showed a broad peak ∼265 nm in the presence of the naphthalene moiety, which became sharper with a distinct blue-shifting of ∼6 nm upon interaction of tRNA with baker's yeast (Fig. S12†). On the other hand, the absorption spectra of probes **4** and **5** upon binding with tRNA from baker's yeast show negligible change (Fig. S13†). Probe **1** shows almost insignificant fluorescence enhancement when exposed to F^–^, I^–^, double-stranded (ds) DNA, single-stranded (ss) DNA, glucose, heme, UTP, TTP, ATP, GTP and CTP (Fig. 2c), indicating the high selectivity of naphthalene-based probe **1** towards recognition of tRNA and denatured tRNA over other anions and nucleotides.

Fluorescence titration experiments of probes **1–3** upon binding with tRNA from baker's yeast and RNA from *torula* yeast were conducted to investigate the binding phenomenon (Fig. S14–S19†). Probes **1–3** display a 1 : 1 binding stoichiometry with tRNA from baker's yeast and RNA from torula yeast (the concentration of RNA was determined spectrometrically and expressed as the molarity of phosphate groups),^26^ suggesting that the imidazolium/triethyl amino group of each probe molecule binds to each phosphate unit of the RNA backbone, through primary electrostatic interaction. Simultaneously, this allows the naphthalene moiety of each probe molecule to have secondary interactions with the stacking nucleobases of RNA. All these observations are further supported by the fact that excimer formation arises from interstitial π–π stacking^22^ between naphthalene moiety and each stacking pair of RNA.^6^ Only one imidazolium group might be involved in binding as evident from the 1 : 1 stoichiometry (Fig. S15 and S18†) in the case of probe **2**. Binding constants^28^,^29^ (∼10^4^ M^–1^) and detection limits^30^ (∼8 × 10^–6^ M) of probes **1–3** were calculated and are summarized in Table 1. Fluorescence titration results were also subjected to a Scatchard plot to calculate binding constants (Fig. S14–S19† and Table 1).^31^ The results are almost comparable to the binding constants calculated based on 1 : 1 binding stoichiometry between the phosphate group of RNA and probes **1–3** (Table 1). Hence, this strengthens our argument that the imidazolium/triethyl amino group of each probe molecule binds to each ribose-phosphate unit of RNA.^31^

**Table 1:** Results of binding stoichiometries, binding constants and detection limits of probes **1–3** with RNA

Probe	Binding stoichiometry	Binding constant (M^–1^)	Binding constant (M^–1^) (Scatchard plot)	Detection limit (M)
**tRNA from baker's yeast**				
**1**	1 : 1	1.26 ± 0.1 × 10^4^	1.25 ± 0.03 × 10^4^	7.76 × 10^–6^
**2**	1 : 1	1.15 ± 0.1 × 10^4^	1.15 ± 0.02 × 10^4^	7.72 × 10^–6^
**3**	1 : 1	1.12 ± 0.09 × 10^4^	1.14 ± 0.03 × 10^4^	7.51 × 10^–6^

**tRNA from torula yeast**				
**1**	1 : 1	1.03 ± 0.1 × 10^4^	1.04 ± 0.02 × 10^4^	8.10 × 10^–6^
**2**	1 : 1	1.07 ± 0.09 × 10^4^	1.02 ± 0.03 × 10^4^	7.86 × 10^–6^
**3**	1 : 1	1.01 ± 0.08 × 10^4^	1.05 ± 0.02 × 10^4^	7.98 × 10^–6^

**CD results (tRNA from baker's yeast)**				
**1**	1 : 1	0.98 ± 0.08 × 10^4^	0.99 ± 0.02 × 10^4^	8.45 × 10^–6^

**CD results (tRNA from torula yeast)**				
**1**	1 : 1	0.97 ± 0.08 × 10^4^	1.00 ± 0.02 × 10^4^	8.66 × 10^–6^

**Fig. 3:**
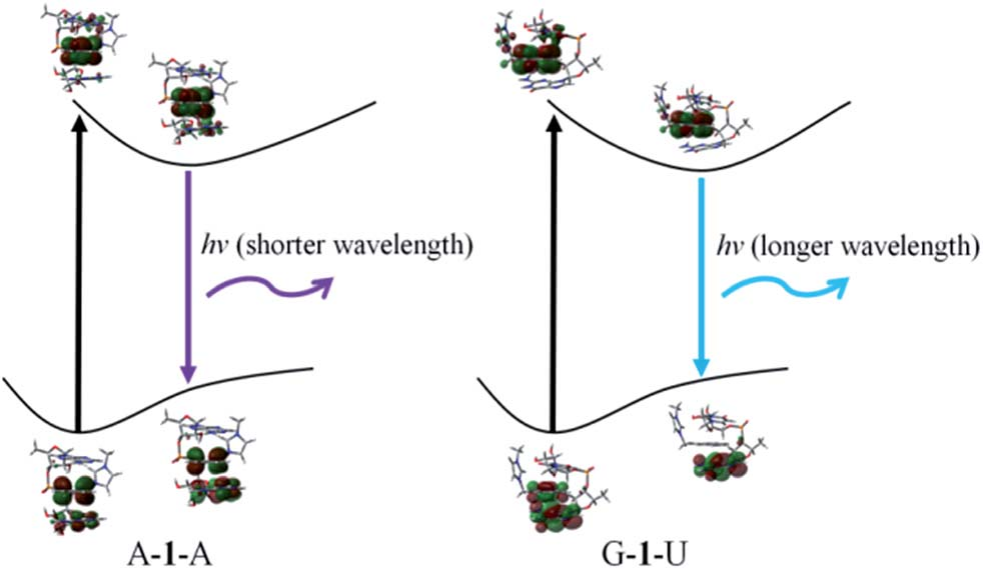
Schematic description of the fluorescence mechanism.

In order to confirm the fluorescence result, we carried out DFT and TD-DFT calculations^32^ on intercalation model systems in which probe **1** is sandwiched between two nucleobases that are connected by a ribose-phosphate backbone (Table S1†). In the ground state, all the structures have perfect triple stacking with π–π interactions. The molecular orbitals (MOs) responsible for the vertical excitation (absorption), mostly the highest occupied MOs (HOMOs) and the lowest unoccupied MOs (LUMOs), are delocalized over the naphthalene and one of the bases. Due to the large spatial overlaps between the HOMOs and LUMOs and the similar structures, no significant difference is observed in wavelength and oscillator strength for absorption between different structures with an intercalated naphthalene moiety. However, the rotation of either one of the two bases or the naphthalene moiety leads to breaking of the spatial overlap between HOMO and LUMO at the 1^st^ excited state minimum, which is essential for de-excitation and fluorescence. This is followed by wide-ranging wavelength values for the 1^st^ excited state optimized structures; the values span from 393 to 503 nm (Fig. 3 and S22†). The structures can be classified in terms of oscillator strength. Fig. S20 and S22† explain that A–**1**–A, A–**1**–C and A–**1**–G have the delocalized HOMOs over one of the bases and the naphthalene moiety, resulting in significant overlap with LUMOs mostly localized on the naphthalene moiety which showed high oscillator strength. On the other hand, other structures present smaller oscillator strength due to localized HOMOs and LUMOs either on naphthalene or one of the bases, so-called charge-transfer (CT) de-excitation. The exceptions are C–**1**–G and C–**1**–U: oscillator strength <0.01 for the former with delocalized HOMO and localized LUMO, but >0.01 for the latter even with CT excitation. The data could be merged together to reproduce the fluorescence result. Considering both Doppler broadening for the finite width to the spectral lines and the number of each stacking pair, the theoretical fluorescence matches experiment well (Fig. 2d and ESI†). This strongly indicates that not only specific stacking pairs but all the intercalation structures are involved in the fluorescence ranging 425–450 nm.

### 2.2. Circular dichroism (CD) studies

**Fig. 4:**
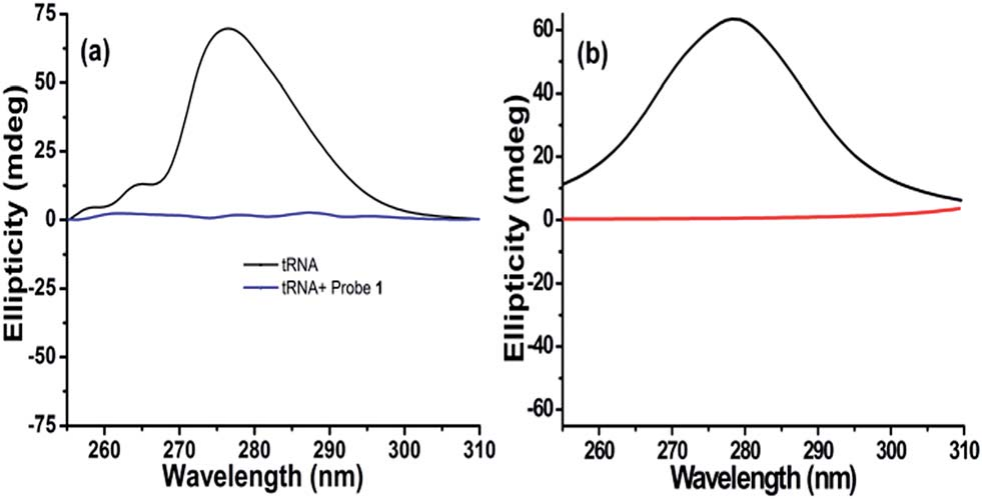
Experimental and theoretically calculated CD data. (a) CD of tRNA from baker's yeast (2 mM) with and without probe **1** (4.5 equiv.). HEPES buffer pH = 7.4. (b) Computed CD results of the tRNA fragment with 10 nucleotides: helical structure without probe **1** (black) and stretched structure with probe **1** (red).

To obtain insight into the conformational change of the RNA structure and binding mechanism upon interaction, probe **1** was selected for circular dichroism (CD) studies.^33^ tRNA from baker's yeast (1 mM) shows positive ellipticity centred at 276.5 nm (Fig. 4a and S23†) while RNA from *torula* yeast shows positive ellipticity centred at 290.5 nm (Fig. S23†) (due to the stacking interactions between the stacking pairs and the helical structure that provide an asymmetric environment for the bases). Addition of probe **1** into tRNA from baker's yeast and RNA from *torula* yeast solution results in a decrease in ellipticity until it becomes almost zero (Fig. 4a and S23†).^34^ CD titration of tRNA from baker's yeast and RNA from torula yeast shows 1 : 1 stoichiometry and the binding constant is calculated assuming 1 : 1 binding stoichiometry (Fig. S24 and S25†). CD titration results are also subjected to Scatchard plot to calculate the binding constants (Fig. S24 and S25†) and the outcomes are comparable to those calculated based on 1 : 1 binding stoichiometry between the phosphate group of RNA and probes **1–3** (Table 1). Thus, this strengthens our argument that the imidazolium/triethyl amino group of each probe molecule binds to each ribose-phosphate unit of RNA.^31^ Based on these observations, we propose that interaction of probe **1** causes tRNA from baker's yeast and RNA from *torula* yeast to unfold their secondary structures, exploiting strong binding between RNA and probe **1**.^35^,^36^ The phenomenon has been supported by fluorescence studies where tRNA and denatured tRNA with probe **1** give almost the same response (Fig. 2c). Fluorescence (Fig. S14–S19e and f†) and circular dichroism (Fig. S24 and S25e and f†) results were also subjected to neighbor exclusion model (proposed by Schellman and Reese) and a cut off of experimental points was observed well below *θ* = 0.5 indicating that the neighbour exclusion principle is upheld in RNA recognition by probes **1–3**.^37^

The computed CD result for the model, shown in Fig. 4b, further confirms our speculation that the stretched RNA structure is responsible for the flat signal in CD experiments. RNA used here is only a local part from the entire tRNA of baker's yeast with a limited number of nucleotides, showing that the wavelength at the maximum ellipticity (∼278 nm) is very close to the experimental result (Fig. 4b). Moreover, the signal for the stretched RNA structure with probe **1** is almost flat as in Fig. 4a. Therefore, we are convinced that the CD results can be attributed to the involvement of probe molecules in the stretch of RNA, where probe molecules are intercalated into every base stacking site, violating the neighbor-exclusion principle. This argument is consistent with the basic insight of hydrophobicity: all the hydrophobic naphthalene fluorophores try to avoid exposure to hydrophilic environment due to lack of attraction with hydrophiles. There are only two possibilities for naphthalene to escape the hydrophilic environment, either aggregation of the probe molecules or intercalation in between nucleobases both using π–π interaction. However in this case, the charged moiety (imidazolium or triethyl amino group) prevents the former because of its solubility in water.

### 2.3. ^1^H NMR studies

Probe **1** was selected in order to monitor the physical interaction through ^1^H NMR experiment. tRNA (GCGCGCGCGC) and tRNA (AUAUAUAUAU) were used to investigate which nucleobase is responsible for the interaction with the naphthalene moiety of probe **1** (see ESI† for details). A 2D NOESY experiment of probe **1** with tRNA from baker's yeast was also recorded in order to investigate the proposed binding pattern. The relatively weak NOE correlation between the naphthalene moiety of probe **1** and the nucleobases of RNA reveals that the naphthalene moiety is in close vicinity to the nucleobases of RNA (Fig. S26†). Downfield shifts associated with splitting of naphthalene protons and upfield shifts of RNA protons (Fig. S27 and S28†) suggest that each nucleobase of RNA is involved in π–π stacking interactions with the naphthalene moiety of probe **1** causing excimer formation and fluorescence with a broad peak centred around 425–450 nm. This was further strengthened by 1 : 1 binding stoichiometry of probe **1** with the phosphate groups of RNA. Fluorescence, circular dichroism and ^1^H NMR data demonstrate that every stacking pair is involved in intercalation and fluorescence, in contrast to the previous conjecture that one naphthalene based cyclophane binds to one RNA molecule.^17^

### 2.4. Molecular dynamics (MD) simulations

**Fig. 5:**
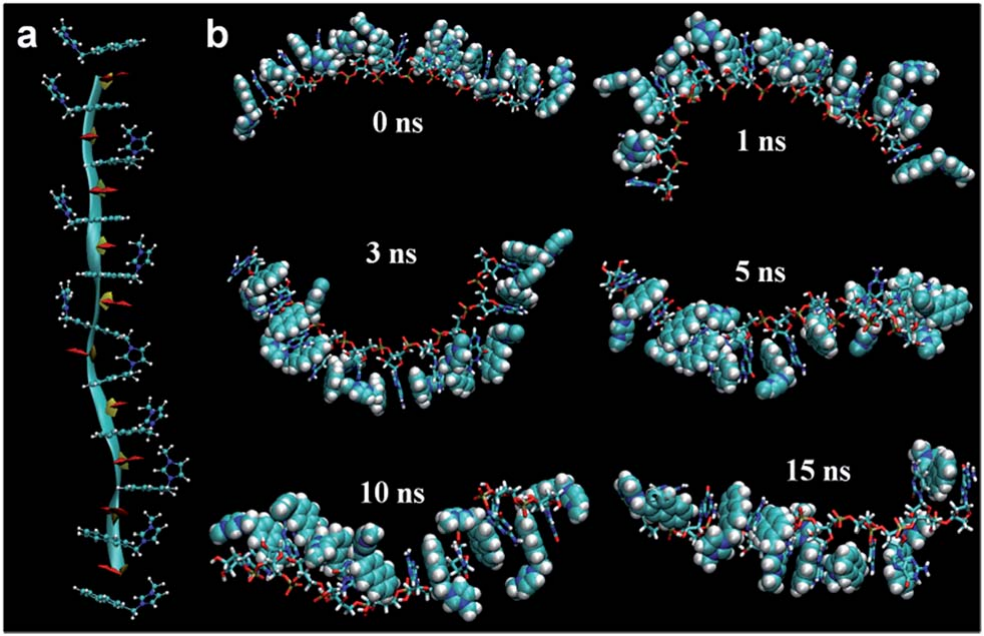
MD simulation results (a) schematic description of tRNA fragment of 10 nucleotides interacting with probe **1**: naphthalene moieties at intercalation sites and imidazoium moieties interacting with phosphate backbone and 2′-hydroxyl group (2′-OH) of ribose (ribbon: phosphate backbone; ball-and-stick: probe **1**; yellow: ribose; red: nucleobase). (b) Snapshots of 15 ns MD simulation in NPT ensemble of the corresponding model (stick: RNA; vdW: probe **1**). Water molecules are removed for clarity.

Furthermore, our MD simulation results support our proposal about unfolding of RNA due to intercalation of probes. We performed MD simulations for RNA with 10 nucleotides in which the naphthalene moiety in probe **1** is located at every intercalation site. The majority of naphthalene moieties in the structure, where some imidazolium moieties interact with phosphate and the others interact with ribose's 2′-OH, maintain the initial intercalation form for 15 ns (Fig. 5). On the other hand, if imidazolium moieties interact only with either the phosphate or the 2′-OH of ribose, the structures with intercalating probe molecules become highly unstable, lasting at most for several nanoseconds (Fig. S30 and S31†). Likewise, the MD simulation of the DNA fragment with the same sequence in which intercalated probe molecules interact only with the phosphate backbone shows the dissolution of almost all the intercalation structures within 0.4 ns (Fig. S32†). This is clearly shown from the root-mean-squared distance and deviation data between a probe molecule and stacking nucleobases for RNA-probe and DNA-probe systems (Fig. S33 and S34†). While the probe molecule in the RNA system maintains the intercalation structure even after 20 ns, the one in the DNA system is solvated out within sub-nanoseconds. These results emphasize the difference between DNA and RNA. The lack of 2′-OH in DNA forces imidazolium moieties to interact only with phosphate backbones for at most a few nanoseconds, making the whole intercalation structure unstable. On the other hand, RNA provides two different options for interaction, 2′-OH and the phosphate backbone, directing imidazolium moieties toward the opposite direction for hydrogen bonding. This offers additional stability for intercalation structures, lasting several tens of nanoseconds. Therefore, we can conclude that once the probe molecules are intercalated, they stay where they are in the initial form for several tens of nanoseconds corresponding to the time scale of fluorescence, ∼1–100 ns. Along with the NMR experiment data, this indicates that the intercalation structures are responsible for the fluorescence results.

## 3. Conclusion

We have shown that the neighbor-exclusion principle is violated in RNA by naphthalene based cationic probes. The control experiments demonstrate that only the naphthalene moiety is small enough to be inserted into intercalation sites of RNA, unlike pyrene and anthracene. Furthermore, fluorescence titration, CD and ^1^H NMR experiments infer that not only specific bases but all the stacking pairs are responsible for the fluorescence, having π–π interactions with the probes. The MD simulation results reveal stable intercalation structures where imidazolium moieties interact with the two H-bonding acceptors (the negatively charged oxygen of phosphate and the 2′-OH of ribose) both present in RNA, providing selectivity towards RNA for the cationic moieties of the probes as compared to DNA which only has the phosphate backbone. The computational results support the CD experimental results, suggesting that RNA is stretched by the intercalation of probe molecules. Additionally, we computationally reproduced fluorescence, providing the fluorescence mechanism and supporting the involvement of all intercalation structures in the fluorescence. The breaking of the spatial overlap between HOMO and LUMO at each 1^st^ excited state minimum gives charge transfer driven de-excitations corresponding to fluorescence at 425–450 nm. Overall, we have proposed an effective strategy for RNA recognition, a small fluorophore for facile intercalation with at least one cationic moiety for hydrogen bonding, which is straightforward for both synthesis and further analysis.

## 4. Experimental section

### 4.1. Materials and methods

The synthesis of compounds **1–5** is described in the ESI.† Synthesized compounds (**1–5**) were fully characterized with standard spectroscopic techniques. Imidazole, 2,6-bis(bromomethyl) naphthalene, 2-bromomethyl naphthalene, 9-bromomethyl anthracene, 1-bromomethyl pyrene and triethyl amine were purchased from Aldrich and were used as such. Sodium salts of ATP, GTP, CTP, TTP and UTP, heme, glucose, dsDNA (from Calf Thymus), RNA (from baker's yeast) and RNA (from *torula* yeast) were also purchased from Aldrich and used without further purification. Tetrabutyl ammonium salts of F^–^ and I^–^ were also purchased from Aldrich and used without further purification. tRNA (GCGCGCGCGC) and tRNA (AUAUAUAUAU) were purchased from XIDT and used as such.

#### Fluorometric analysis

Stock solutions of compounds **1**, **2**, **3**, **4**, and **5** (1 mM) were prepared at pH 7.4 in 0.01 M HEPES buffer water mixture and used in the preparation of titration solutions by appropriate dilution up to 10 μM. Aliquots of ATP, GTP, CTP, UTP, TTP, heme, glucose, dsDNA, ssDNA, RNA from baker's yeast, RNA from torula yeast, tRNA (GCGCGCGCGC) and tRNA (AUAUAUAUAU) in 0.01 M HEPES buffer water mixture were then injected into the sample solution through a rubber septum in the cap. dsDNA solution was heated to 90 °C and rapidly cooled at 4 °C in order to denature it.^26^ Similarly tRNA from baker's yeast was also denatured by heating its solution to 90 °C and rapidly cooling at 4 °C.^26^

#### Circular dichroism studies

The CD spectra were collected at room temperature (25 ± 0.2 °C) using a Jasco made J-815 CD spectropolarimeter. Scans were from 350 to 200 nm with a resolution of 1 nm, with data sampling every 5 s. The 1 cm cell contained 1 mM solution of the RNA in 0.01 M HEPES buffer (pH 7.4).^26^ 0.1 M solution of probe **1** was also prepared in 0.01 MHEPES buffer (pH 7.4). CD spectra were then recorded with pure RNA and with addition of specific amount of probe **1**.

#### MD simulations

The atomic coordinates of tRNA of baker's yeast were taken from the Protein Data Bank (PDB) (entry 3EPK: tRNA of baker's yeast entangled with eukaryotic dimethylallyltransferase; Fig. S29a†).^32^ Missing hydrogen atoms were added using the psfgen module implemented in NAMD program.^38^ 10 nucleotides were obtained from this structure (sequenced as AGACGACGCG) and their backbones were stretched for probe molecules to be intercalated in between bases. Ribose groups were patched up by deoxyribose groups for DNA construction. The topology and parameters of probe **1** were constructed using the CHARMM general force field (CGenFF) program after geometry optimization at the M06/6-31G* level.^39^–^41^ The structures are given in Fig. 5 and S30–S32.† The entire structure consisting of the RNA/DNA fragment and probe **1** molecules was then soaked into TIP3P water box. After minimizing the box for 10 heating from 0 to 295 K for 10 ps, we equilibrated the structure in NPT ensemble for 1 ns using the Nose-Hoover Langevin piston pressure control.^42^ Next, we performed MD simulations using the NAMD program and CHARMM36 force field^43^ with periodic boundary conditions and particle-mesh Ewald (PME) full electrostatics.^44^ Coarse PME grid was used to speed up the simulations. By applying Langevin forces the temperature was maintained at 295 K. Van der Waals energies were calculated using cutoff of 12 Å. The MD simulations were performed for 15 ns.

#### (TD-)DFT computations

The single point TD-DFT computations were performed for a structure with probe **1** at the intercalation sites obtained from the previous MD simulation and an original helical structure (from the original PDB file) for theoretical CD. The M06/6-31G* level of theory was employed with conductor polarizable continuum model (CPCM).^45^,^46^

For the in-depth study of the fluorescence mechanism, we performed detailed (TD-)DFT computations further. We acquired some representative base–base stacking fragments including ribose-phosphate backbone from the above mentioned PDB file; we could obtain A–A, A–C, A–G, A–U, C–G, C–U, G–G, G–U and U–U stacking pairs. Then, probe **1** was intercalated in between each stacking base due to its smallest size among the probes we synthesized. To deal with the systems more realistically, some water molecules were added into the first solvation shell around bases along with the CPCM. We optimized the ground and 1^st^ excited states and measured the energies and oscillator strengths. For CD calculations, we used M06/6-31G* level of theory for (TD-)DFT computations since it gives a similar UV-visible absorption spectrum (maximum oscillator strength of 0.91 at wavelength of 215.5 nm) of the probe **1** to the experiment (maximum intensity at wavelength of 224.3 nm).^47^–^50^ All calculations were performed using Gaussian 09 program.^51^

In order to reproduce the fluorescence results, practically it is too time-consuming to compute all the pairs explicitly using the method explained above. Instead, we made approximations; the wavelengths and oscillator strengths of the same stacking nucleobases are the same. Also, one more important point is the Doppler broadening, which brings out a finite width to the spectral lines.^52^ After considering the Doppler broadening, the oscillator strength values obtained from a certain stacking pair were multiplied by the number of the pair in the given RNA structure, which comprises 1 A–A, 6 A–C, 11 A–G, 4 A–U, no C–C, 9 C–G, 10 C–U, 4 G–G, 9 G–U and 3 U–U stacking pairs.

## Supplementary Material

Supplementary information
