# Supplementary material for: Violation of DNA neighbor exclusion principle in RNA recognition
Source: Chem Sci. 2016 Feb 15;7(6):3581–8. doi: 10.1039/c5sc03740a (PMC6007354; doi:10.1039/c5sc03740a)
Supplement: Supplementary file 1 [file SC-007-C5SC03740A-s001.pdf]

Electronic Supplementary Material (ESI) for Chemical Science.
This journal is © The Royal Society of Chemistry 2016

Supporting Information

Violation of DNA Neighbor-Exclusion Principle in RNA Recognition

Muhammad Yousuf,[†,‡,§] Il Seung Youn,[†,‡,§] Jeonghun Yun,[†,‡] Lubna Rasheed,[†] Rosendo Valero,[†] Genggongwo Shi[†,‡] and Kwang S. Kim*[,†]

[†]Department of Chemistry, Ulsan National Institute of Science and Technology (UNIST), Ulsan 689-798, Korea

[‡]Center for Superfunctional Materials, Department of Chemistry, Pohang University of Science and Technology, Pohang 790-784, Korea

[§]These authors contribute equally to this work.

Ulsan National Institute of Science and Technology (UNIST)

Ulsan 689-798, Korea

Phone : + 82-52-217-5410, Fax: +82-52-217-5419

**Email*: kimks@unist.ac.kr

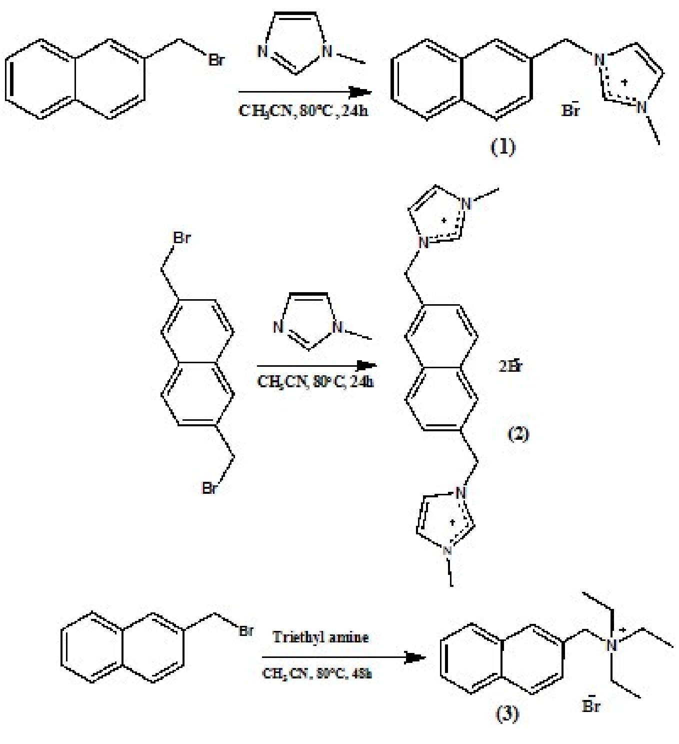

Scheme S1. Synthesis of compounds **1, 2** and **3.**

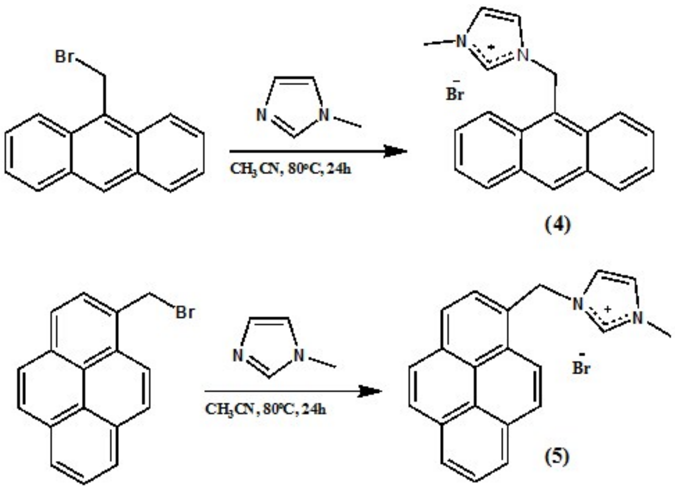

Scheme S2. Synthesis of compounds **4** and **5.**

Synthesis of probe 1

To a solution of 2-bromomethyl naphthalene (0.5 mmol , 110 mg) in dry acetonitrile (25 ml) was added 1-methyl imidazole (1 mmol, 82 μl) and refluxed for 24 hours. The solution was allowed to cool and the solvent was evaporated on roptary evaporated. After exaporation of solvent the product was crystallized out. The obtained product was in 90% yield. ^{1}H NMR (Figure S1) (400 MHz, $(CD_3)_2SO$) 9.373 (s, 1H), 7.956−8.030 (m, 4H, naphthalene ring -C**H**=C**H**-C**H**=), 7.909-7.918 (m, 1H, imidazolium ring -N-C**H**=C**H**-N-), 7.795-7.804 (m, 1H, imidazolium ring -N-C**H**=C**H**-N-), 7.577-7.618 (m, 3H, naphthalene ring -C**H**=C**H**-C**H**=), 5.659 (s, 2H, $-CH_2-$), 3.916 (s, 3H, $-CH_3$); ^{13}C NMR (Figure S2) (100 MHz, $(CD_3)_2SO$, 25° C) δ 35.893, 51.987, 122.429, 123.985, 125.738, 126.716, 126.739, 127.543, 127.672, 127.855, 128.302, 128.750, 132.286, 132.695, 136.785. HRMS (EI, m/z): $[M-Br]^+$ calc.: 302.04; found: 302.01; Anal. Calcd for $C_{15}H_{15}BrN_2$: C, 59.42; H, 4.99; N, 9.24, Found: C, 59.39; H, 4.97; N, 9.27.

Synthesis of probe 2

To a solution of 2,6-bis(bromomethyl) naphthalene (0.5 mmol , 157 mg) in dry acetonitrile (25 ml) was added 1-methyl imidazole (2 mmol, 165 μl) and refluxed for 24 hours. The solution was allowed to cool and white precipitates formed were collected. The obtained product was in 95% yield. ^{1}H NMR (Figure S3) (400 MHz, $(CD_3)_2SO$) 9.337 (s, 2H), 8.010−8.032 (m, 4H, naphthalene ring -C**H**=C**H**-C**H**=), 7.863-7.871 (m, 2H, imidazolium ring -N-C**H**=C**H**-N-), 7.783-7.792 (m, 2H, imidazolium ring -N-C**H**=C**H**-N-), 7.607-7.633 (m, 2H, naphthalene ring -C**H**=C**H**-C**H**=), 5.652 (s, 4H, $-CH_2-$), 3.910 (s, 6H, $-CH_3$); ^{13}C NMR (Figure S4) (100 MHz, $(CD_3)_2SO$, 25° C) δ 35.911, 51.875, 122.409, 124.048, 126.461, 127.349, 128.874, 132.447, 133.138, 136.848. HRMS (EI, m/z): $[M-Br]^+$ calc.: 476.02; found: 475.98; Anal. Calcd for $C_{20}H_{22}Br_2N_4$: C, 50.23; H, 4.64; N, 11.72, Found: C, 50.31; H, 4.61; N, 11.77.

Synthesis of probe 3

To a solution of 2-bromomethyl naphthalene (0.5 mmol , 110 mg) in dry acetonitrile (25 ml) was added triethyl amine (1 mmol, 139 μl) and refluxed for 24 hours. The solution was allowed to cool and the solvent was evaporated on roptary evaporated. After exaporation of solvent the waxy product was crystallized out. The obtained product was in 88% yield. ^{1}H NMR (Figure S5) (400 MHz, $(CD_3)_2SO$) 7.632−8.187 (m, 7H, naphthalene ring -C**H**=C**H**-C**H**=), 4.731 (s, 2H, $-CH_2-$), 1.38-1.409 (m, 6H, $-CH_2-$), 1.220-1.257 (t, 9H, $-CH_3$); ^{13}C NMR (Figure S6) (100 MHz, $(CD_3)_2SO$, 25° C) δ 7.640, 52.134, 59.638, 125.369, 126.803, 127.524, 127.554, 128.351, 128.487, 128.943, 132.478, 132.843, 133.192. HRMS (EI, m/z): $[M-Br]^+$ calc.: 321.11; found: 321.08; Anal. Calcd for $C_{17}H_{24}BrN$: C, 63.35; H, 7.51; N, 4.35, Found: C, 63.44; H, 7.46; N, 4.38.

Synthesis of probe 4

To a solution of 9-bromomethyl anthracene (0.5 mmol , 135 mg) in dry acetonitrile (25 ml) was added 1-methyl imidazole (1 mmol, 82 μl) and refluxed for 24 hours. The solution was allowed to cool and the solvent was evaporated on roptary evaporated. After exaporation of solvent the product was crystallized out. The obtained product was in 93% yield. ^{1}H NMR (Figure S7) (400 MHz, $(CD_3)_2SO$) 8.78-8.88 (m, 1H), 8.497−8.520 (m, 2H, anthracene ring -C**H**=C**H**-C**H**= and imidazolium ring -N-C**H**=C**H**-N-), 8.254−8.275 (m, 2H, anthracene ring -C**H**=C**H**-C**H**= and imidazolium ring -N-C**H**=C**H**-N-), 7.637-7.775 (m, 7H, anthracene ring -C**H**=C**H**-C**H**=), 6.256 (s, 2H, $-CH_2-$), 3.778 (s, 3H, $-CH_3$); ^{13}C NMR (Figure S8) (100 MHz, $(CD_3)_2SO$, 25° C) δ 35.787, 44.817, 122.460, 123.416, 123.530, 123.712, 125.571, 127.764, 129.372, 130.078, 130.610, 131.072, 135.989. HRMS (EI, m/z): $[M-Br]^+$ calc.: 352.06; found: 352.01; Anal. Calcd for $C_{19}H_{17}BrN_2$: C, 64.60; H, 4.85; N, 7.93, Found: C, 64.75; H, 4.93; N, 7.90.

Synthesis of probe 5

To a solution of 1-bromomethyl pyrene (0.5 mmol , 148 mg) in dry acetonitrile (25 ml) was added 1-methyl imidazole (1 mmol, 82 μl) and refluxed for 24 hours. The solution was allowed to cool and the solvent was evaporated on roptary evaporated. After exaporation of solvent the product was crystallized out. The obtained product was in 86% yield. ^{1}H NMR (Figure S9) (400 MHz, $(CD_3)_2SO$) 9.237 (s, 1H), 8.162−8.537 (m, 9H, pyrene ring -C**H**=C**H**-C**H**=), 7.921 (s, 1H, imidazolium ring -N-C**H**=C**H**-N-), 7.780 (s, 1H, imidazolium ring -N-C**H**=C**H**-N-), 6.270 (s, 2H, $-CH_2-$), 3.862 (s, 3H, $-CH_3$); ^{13}C NMR (Figure S10) (100 MHz, $(CD_3)_2SO$, 25° C) δ 35.884, 49.906, 122.359, 122.617, 123.657, 123.915, 124.089, 125.205, 125.789, 125.986, 126.669, 127.246, 127.344, 127.944, 128.164, 128.589, 128.794, 130.129, 130.675, 131.487, 136.692. HRMS (EI, m/z): $[M-Br]^+$ calc.: 376.06; found: 376.01; Anal. Calcd for $C_{21}H_{17}BrN_2$: C, 66.85; H, 4.54; N, 7.43, Found: C, 66.75; H, 4.48; N, 7.49.

1. **NMR spectral analysis:**

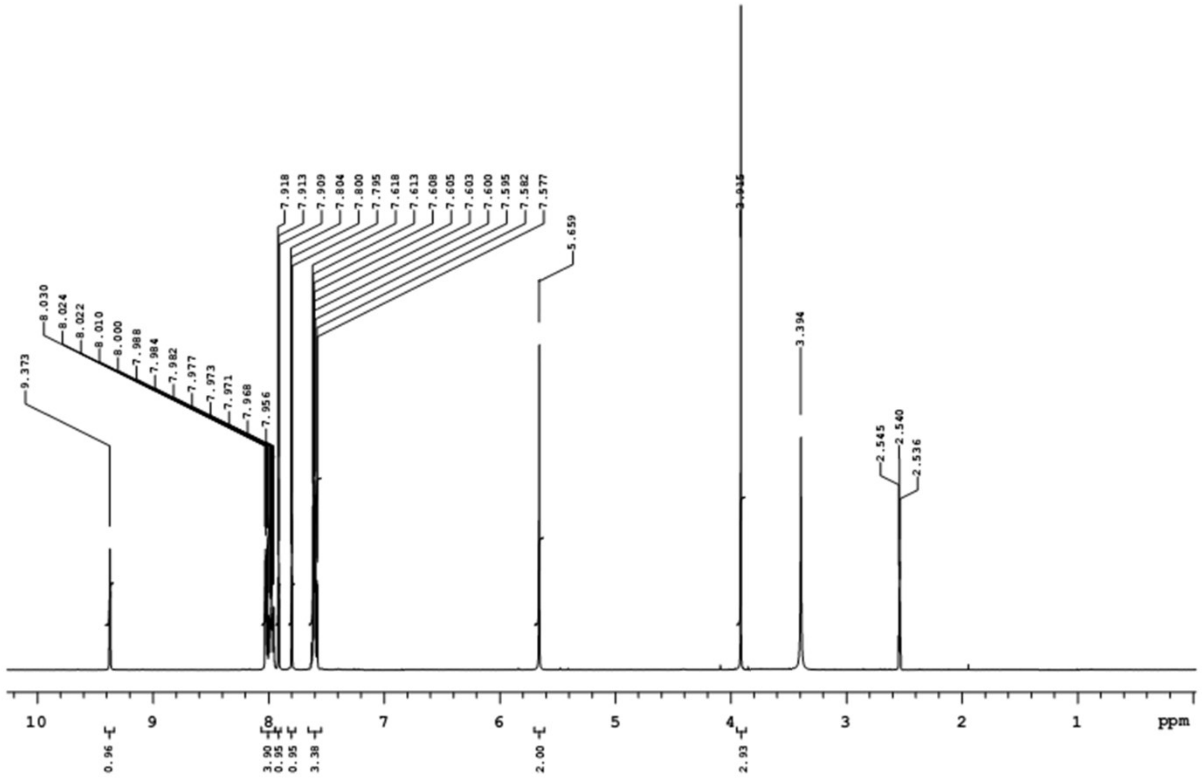

Figure S1. ^{1}H NMR spectrum of compound **1** in DMSO-d_6.

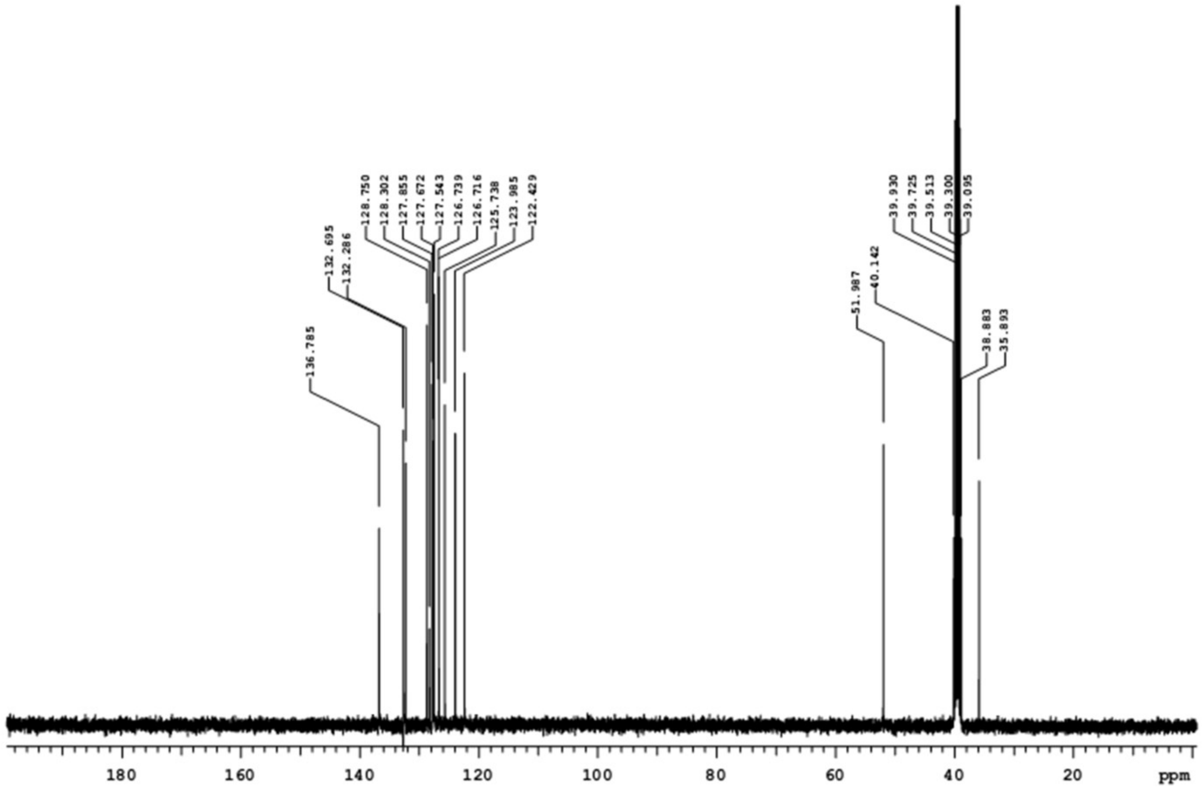

Figure S2. ^{13}C NMR spectrum of compound **1** in DMSO-d_6.

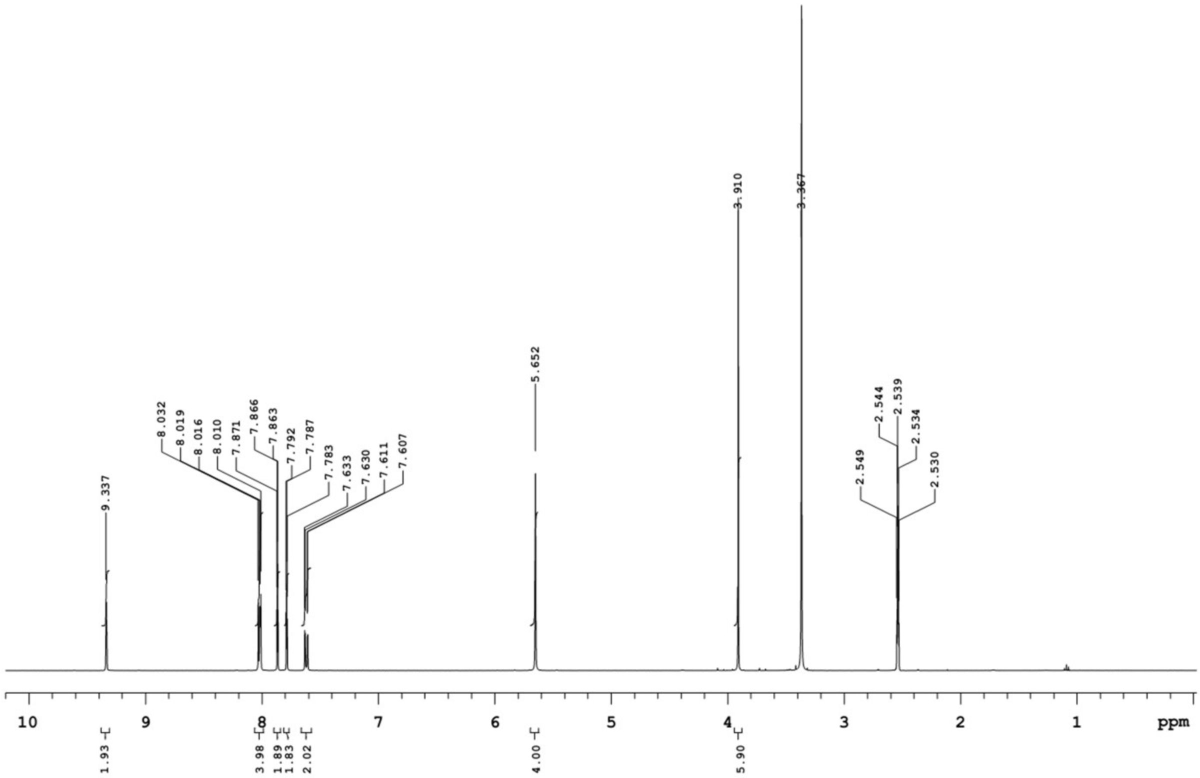

Figure S3. ^{1}H NMR spectrum of compound **2** in DMSO-d_6.

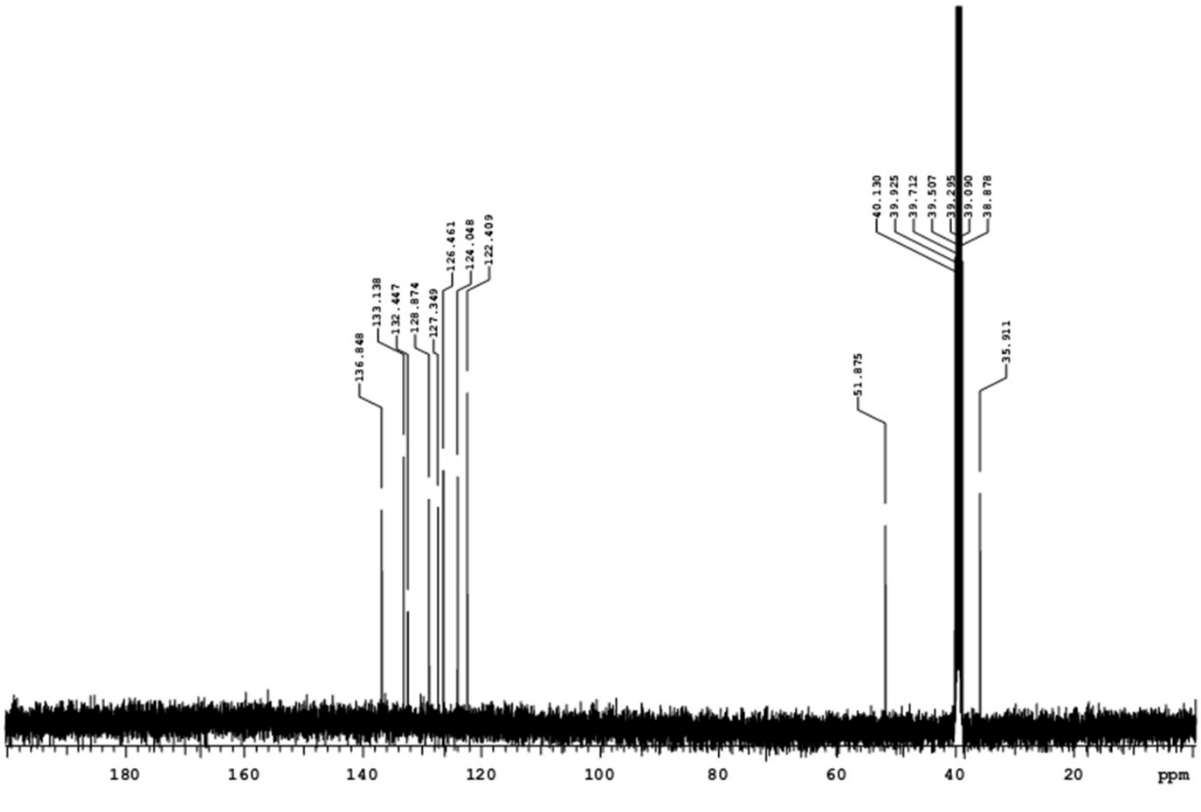

Figure S4. ^{13}C NMR spectrum of compound **2** in DMSO-d_6.

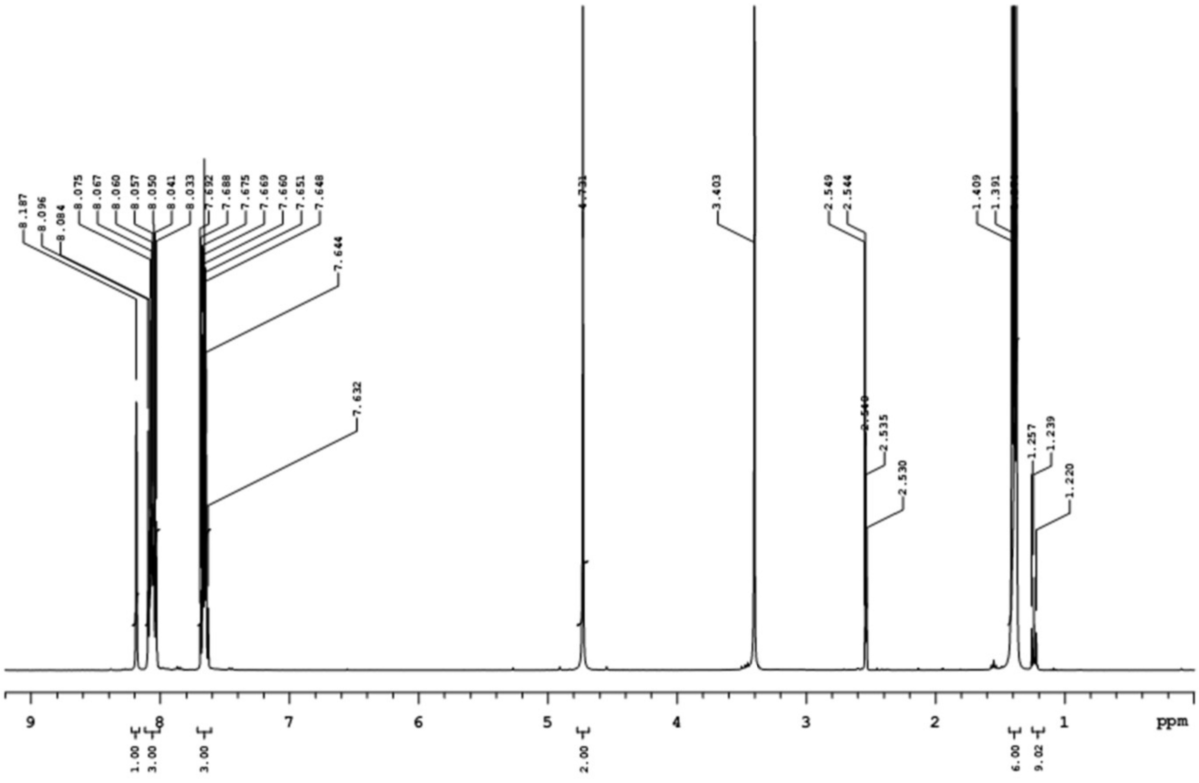

Figure S5. ^{1}H NMR spectrum of compound 3 in DMSO-d_6.

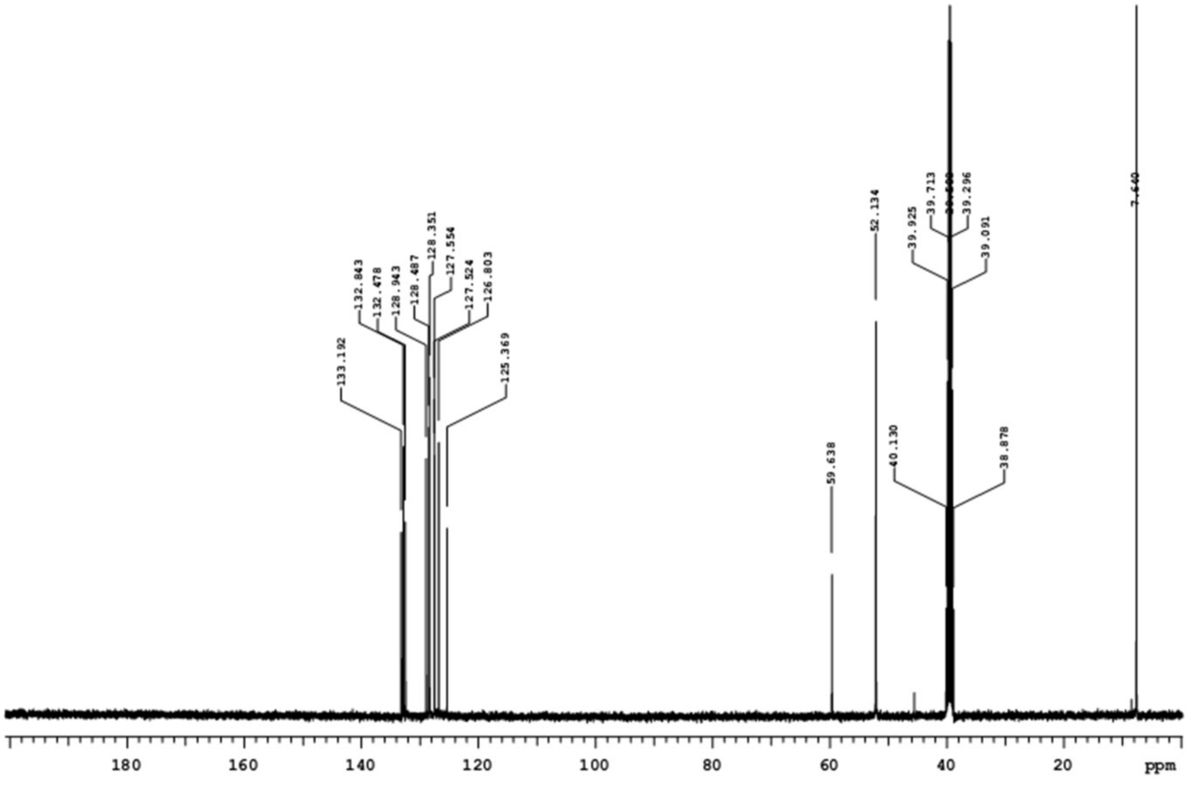

Figure S6. ^{13}C NMR spectrum of compound **3** in DMSO-d_6.

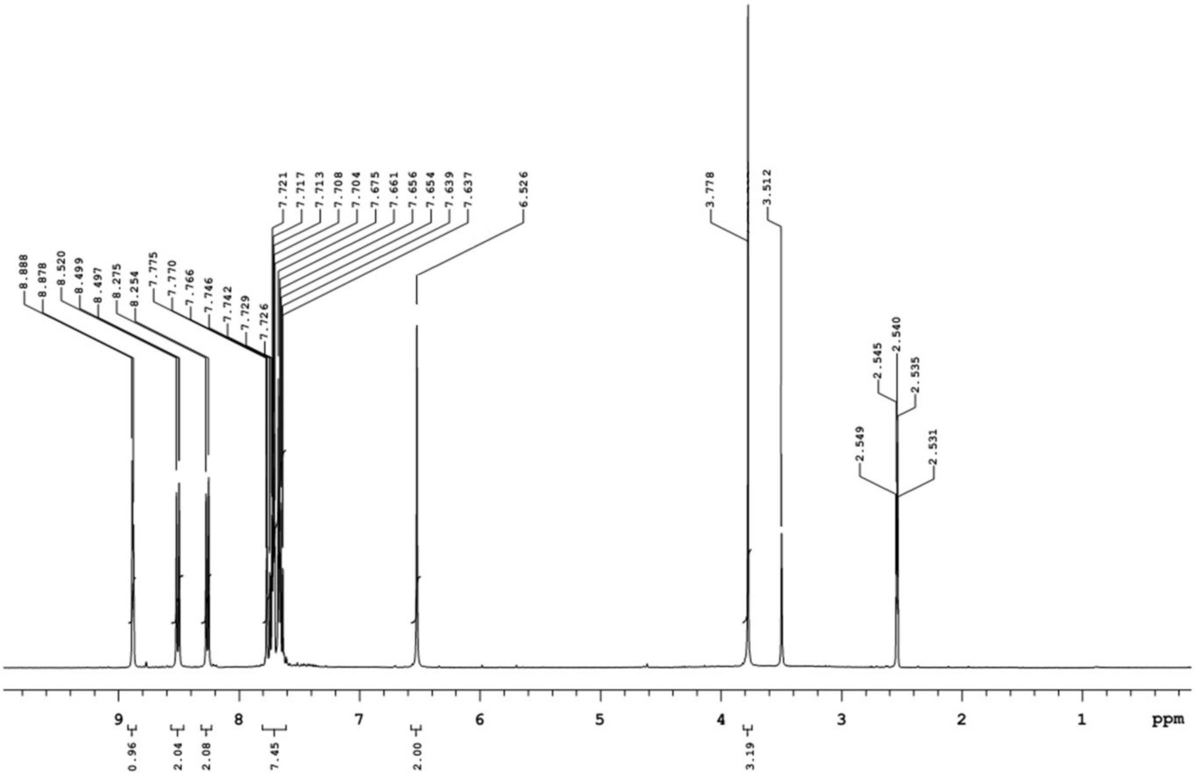

Figure S7. ^{1}H NMR spectrum of compound **4** in DMSO-d_6.

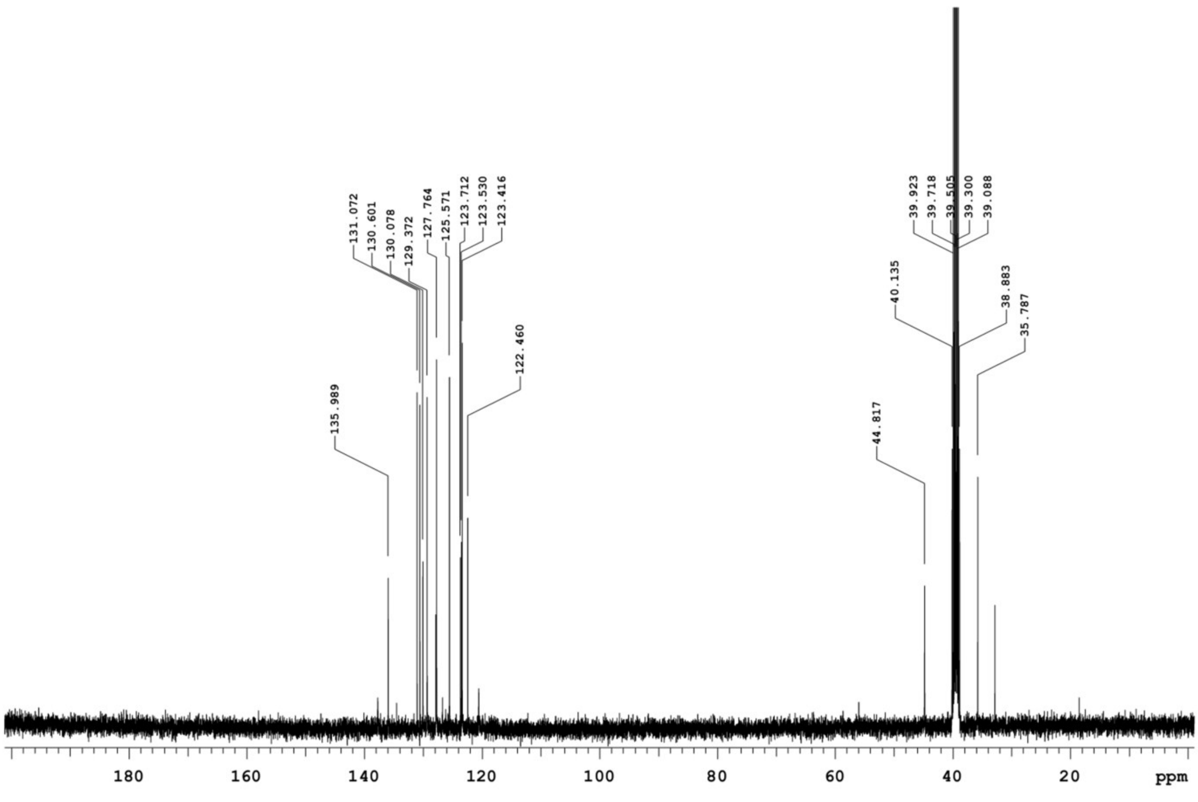

Figure S8. ^{13}C NMR spectrum of compound **4** in DMSO-d_6.

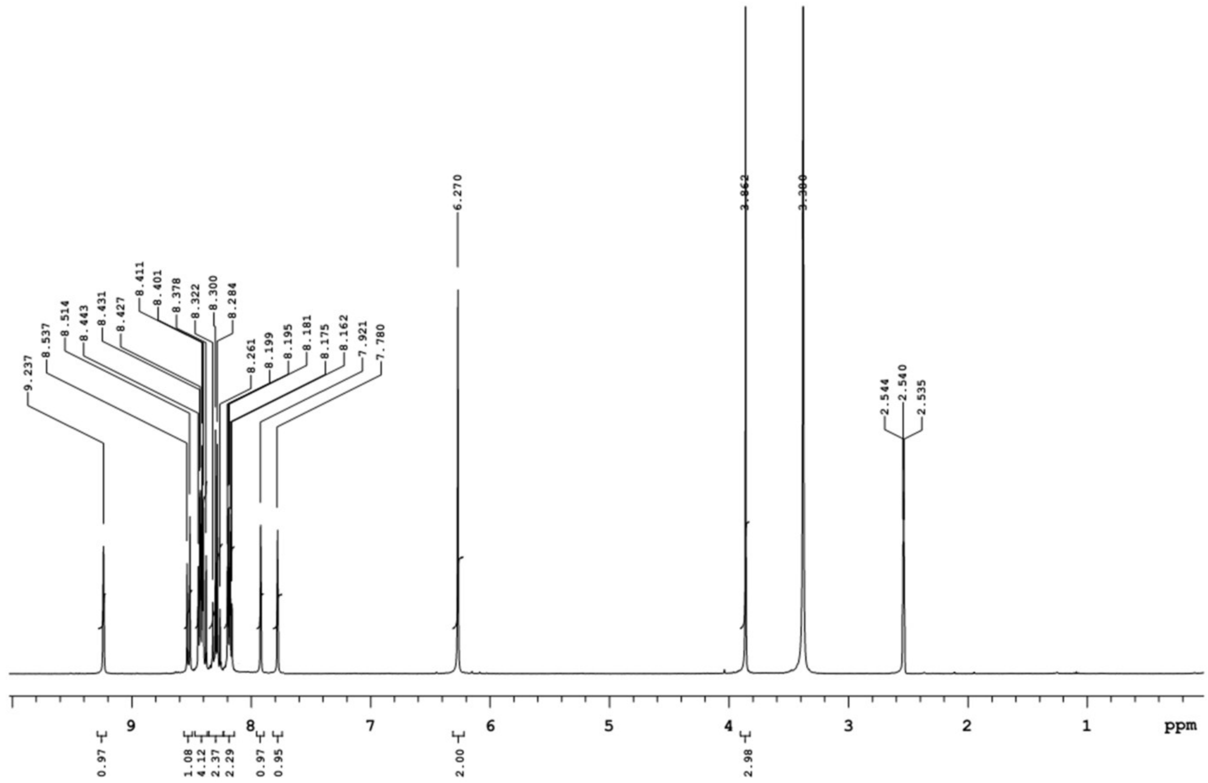

Figure S9. ^{1}H NMR spectrum of compound **5** in DMSO-d_6

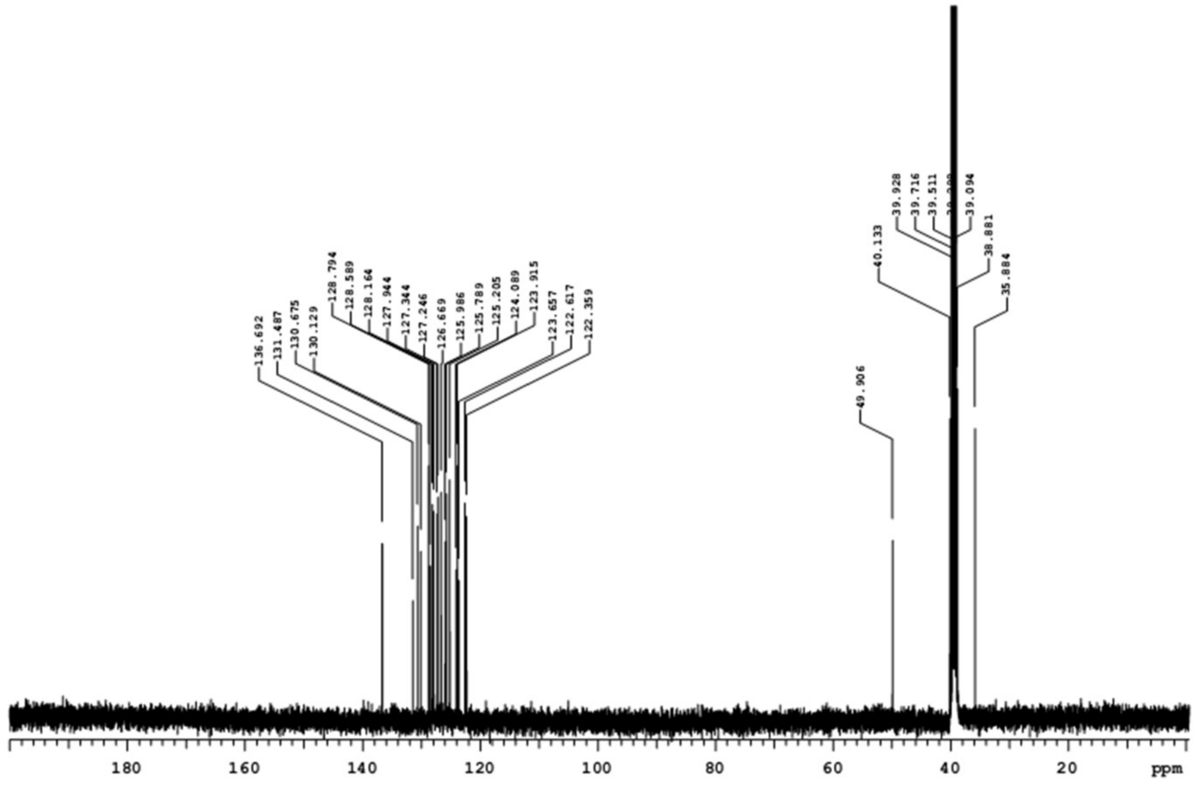

Figure S10. ^{13}C NMR spectrum of compound **5** in DMSO-d_6

2. Experimental techniques

Compounds were fully characterized with standard spectroscopic techniques. Microanalyses were performed on a Carlo 1102 elemental analysis instrument. Electronic absorption (UV-Vis) spectra were recorded using a Shanghai 756 MC UV-Vis spectrometer.^{1}H NMR and ^{13}C NMR, spectra were performed on a varian (400 MHz) spectrometer at 298 K. High resolution mass spectra were obtained on a Micromass Platform II mass spectrometer. Fluorescence studies were carried out on Shimadzu RF-5301 PC spectrofluorophotometer at 298 K. Circular dichroism (CD) spectra was recorded on Jasco made J-815 CD spectropolarimeter.

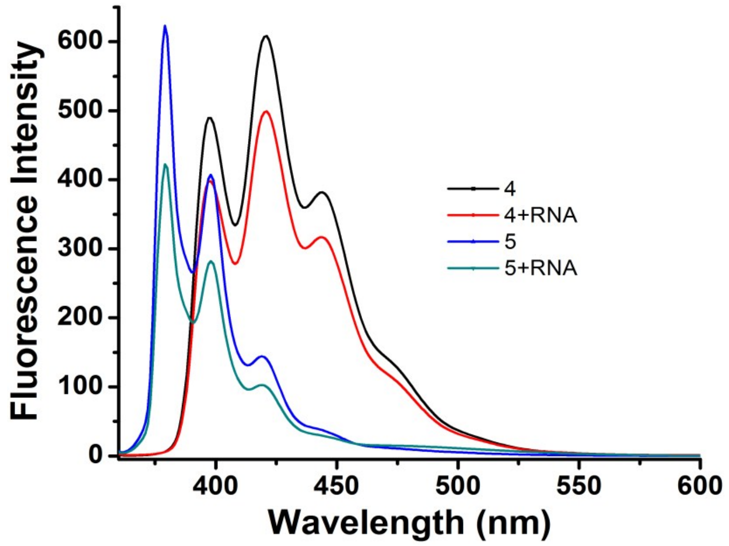

Figure S11. Fluorescence of probes **4, 5** (10 μM) with 10 equiv. tRNA from baker's yeast at pH 7.4 (0.01 M HEPES buffer, 25°C).

3. Quantum Yield Measurements

Absolute fluorescence quantum yields for **1**, **2**, **3**, **4**, and **5** were measured using Shanghi 756 MC UV-Vis spectrometer and Shimadzu RF-5301 PC spectrofluorophotometer. Naphthalene as standard was used for quantum yield measurement of probes **1**, **2**, and **3** while anthracene standard was used for quantum yield measurement of probes **4** and **5**. Typically, the quantum yield was obtained by integrating the photons emitted by **1** up to 600 nm and calculated according to following formula.[1]

$$\mathbf{\Phi(Un) = [FLI\ (Un)/Abs(Un)]\times[Abs(Std)/FLI\ (Std)]\times[\{n(Un)/n(Std)\}^2]\times\ \Phi(Std)}$$

Where:

$\mathbf{\Phi}_{(Un)}$ = Quantum yield of unknown

$\mathbf{\Phi}_{(Std)}$ = Quantum yield of standard

$FLI_{(Un)}$ = Fluorescence of unknown

$FLI_{(Std)}$ = Fluorescence of standard

$Abs_{(Un)}$ = Absorbance of unknown

$Abs_{(Std)}$ = Absorbance of standard

$n_{(Un)}$ = Refractive index of solvent in which fluorescence and absorbance of unknown sample has been carried out.

$n_{(Std)}$ = Refractive index of solvent in which fluorescence and absorbance of standard sample has been carried out.

4. UV/Vis Spectral Analysis

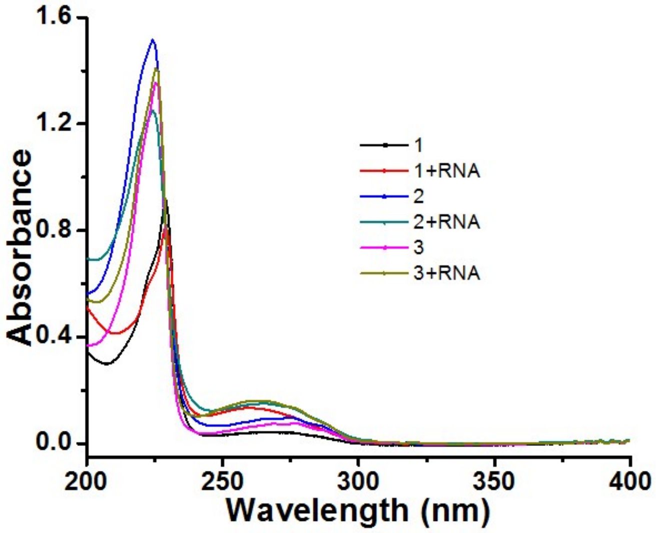

Figure S12. Absorption spectra of **1, 2** and 3 (10 μM) upon addition of RNA (10 equiv) at pH 7.4 (0.01M HEPES buffer, 25°C).

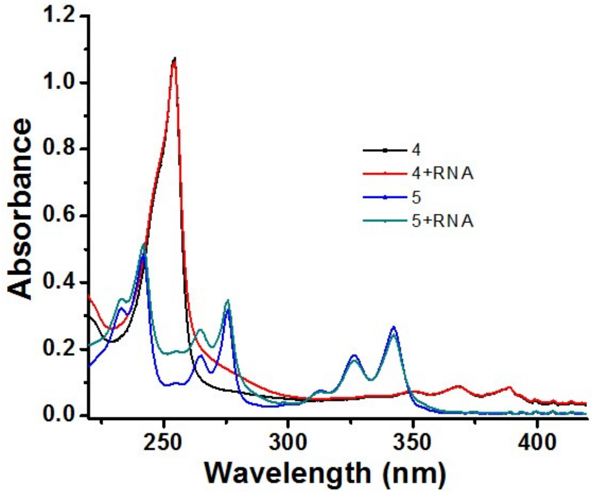

Figure S13. Absorption spectra of **4, and 5** (10 μM) upon addition of RNA (10 equiv) at pH 7.4 (0.01M HEPES buffer, 25°C).

5. Fluorometric Analysis

All spectrofluorimetric titrations were performed as follows. Stock solution of compound (**1**, **2**, **3**, **4**, and **5**) (1mM) was prepared at pH 7.4 in 0.01 M HEPES buffer water mixture and used in the preparation of titration solution by appropriate dilution up to 10 μM. Aliquots of ATP, GTP, CTP, UTP, TTP, Heme, Glucose, dsDNA, ssDNA and RNA from baker's yeast in 0.01 M HEPES buffer water mixture was then injected into the sample solution through a rubber septum in the cap. The concentration of the RNA was also determined from the extinction coefficient at 260 nm (ε_{260} =9250 $M^{-1}cm^{-1}$) using a UV-Visible spectrophotometer in 10 mM phosphate buffer water mixture and were then injected into the sample solution.[1] The concentrations of DNA solutions in 10 mM phosphate buffer (pH 7.4) were determined by using the average value of 6600 $M^{-1}cm^{-1}$ for the extinction coefficient of a single nucleotide at 260 nm.[2] dsDNA solution was heated up-to 90°C and rapidly cooled at 4°c in order to denature it.[2] Similarly tRNA from baker's yeast was also denatured by heating its solution up-to 90°C and rapidly cooled at 4°C.[2] To account for dilution effects, these stock solutions also contained the receptors at its initial concentration. The sample solutions were magnetically stirred for 1 minute after each addition before rescaning. This process was repeated until the change in fluorescence intensity became insignificant. Binding constants K_a for RNA with Probes **1-3** were derived from the plots of F/F_0 vs [RNA] by assuming one site model using Origin Lab 8.0 (Figures S14-19 and S24-25).[3] Results reported in the main text are the average of at least two independent titrations. Emission spectrum was measured by keeping slit width = 5 nm and λ_{exc}= 350 nm.

Binding constant was also determined from scatchard plot (Figures S14-19 and S24-25) based on fluorescence titration and CD and the results are compared in Table 1. The concentration of the RNA-bound ligand was calculated according to equation 1.

$$c\text{b} = c\text{L} \times (F - F\text{f}/F\text{b} - F\text{f}) \qquad \text{eq. 1}$$

cL = Bulk concentration of the ligand

Ff = Fluorescence of the free ligand

Fb = Fluorescence of the bound ligand

F = Fluorescence at a given ligand-to-RNA ratio

c = The concentration of the unbound ligand

r = Ratio of bound ligand molecules per RNA base pair (determined according to equation 2 and 3)

$$c = c\text{L} - c\text{b} \qquad \text{eq. 2}$$

$$r = c\text{b}/c\text{RNA} \qquad \text{eq. 3}$$

The data were given as Scatchard plots, r/c *vs.* r, and numerically fitted to the model of McGhee and von Hippel (eq. 4) using nonlinear curve fitting using Origin Lab 8.0,[4] to deduce the binding constant.

$$\frac{r}{c} = K(1 - nr)\left[\frac{1 - nr}{1 - (n-1)r}\right]^{(n-1)} \qquad \text{eq. 4}$$

The experimental data was also subjected to Schellman and Reese model for verification of neighbor exclusion model (eq. 5 and 6) using nonlinear curve fitting by the aid of Origin Lab 8.0.[5]

$$\frac{\theta}{L} = \frac{K(1-2\theta)^2}{(1-\theta)} \qquad \text{eq. 5}$$

$$L = \frac{\theta(1-\theta)}{K(1-2\theta)^2} \qquad \text{eq. 6}$$

Where;

L = Ligand activity

K = Binding Constant

$\theta = (KL)/(1+KL)$

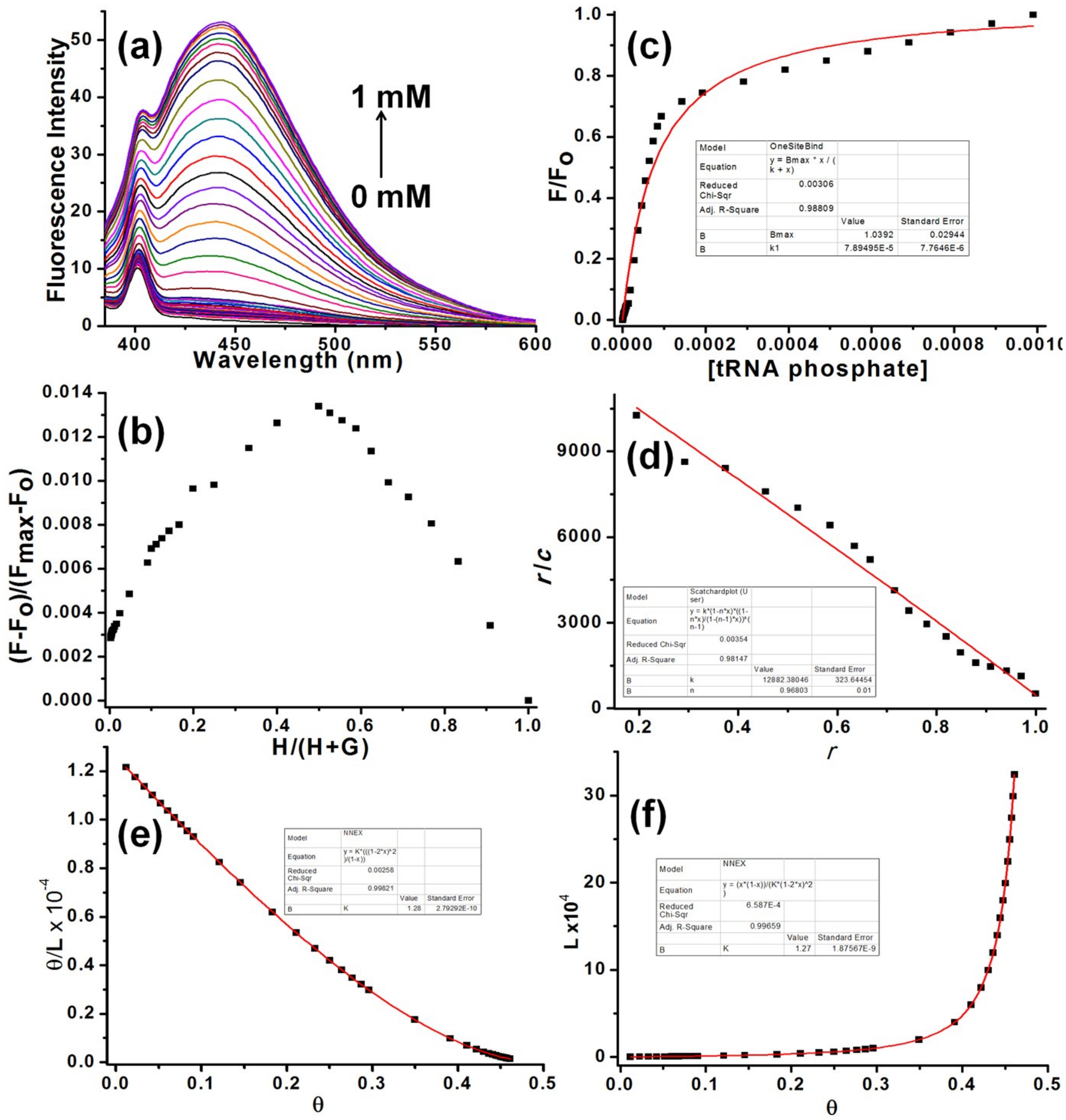

Figure S14. Fluorescence titration of probe **1** with tRNA from Baker's yeast. (a) Emission spectra (excitation at 350 nm) of Probe **1** (10 µM) upon addition of tRNA from Baker's yeast at pH 7.4 (0.01 M HEPES buffer, 25°C). (b) Assessment of the stoichometry of the RNA complex of **1** via Job plot analysis; [**1**] + [RNA] = 10 µM, pH 7.4 (10 mM HEPES buffer), 25ºC. (c) Corresponding binding isotherm of titration. (d) Scatchard plots, *r*/*c* vs *r*; *r* = ligand-to-RNA ratio, obtained from spectrofluorometric titrations of compound **1**-RNA in HEPES buffer (pH 7.4). The experimental data points were fitted to the model of McGhee and von Hippel. (e) Plot of θ/L vs θ, the curve is cut off below 0.5. (f) Corresponding plot of L vs θ showing curve is cut off well below 0.5.

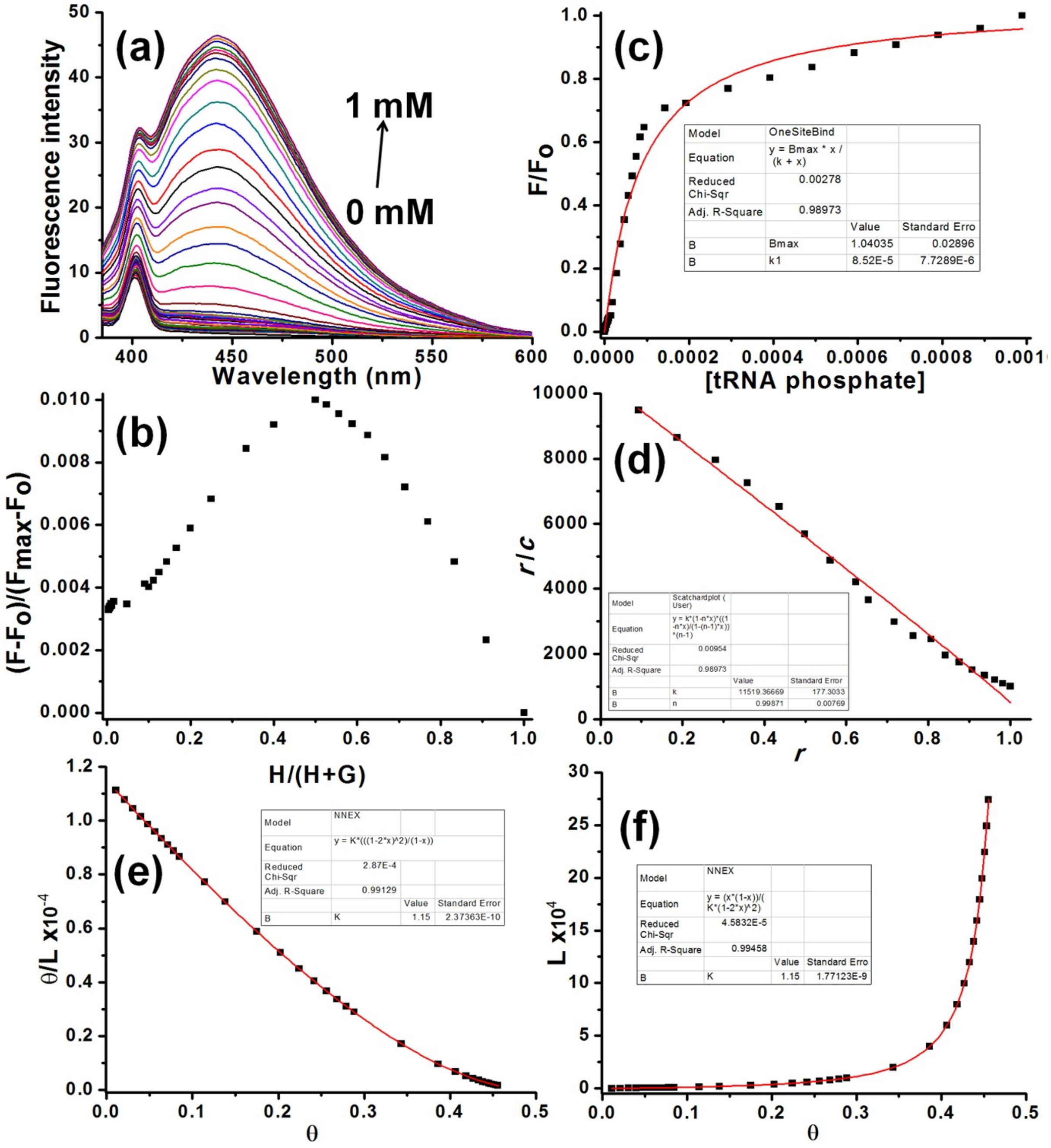

Figure S15. Fluorescence titration of probe **2** with tRNA from Baker's yeast. (a) Emission spectra (excitation at 350 nm) of Probe 2 (10 µM) upon addition of tRNA from baker's yeast at pH 7.4 (0.01 M HEPES buffer, 25°C). (b) Assessment of the stoichiometry of the RNA complex of 2 via Job plot analysis; [2] + [RNA] = 10 µM, pH 7.4 (10 mM HEPES buffer), 25ºC. (c) Corresponding binding isotherm of titration. (d) Scatchard plots, *r*/*c* vs *r*; *r* = ligand-to-RNA ratio, obtained from spectrofluorometric titrations of compound **2**-RNA in HEPES buffer (pH 7.4). The experimental data points were fitted to the model of McGhee and von Hippel. (e) Plot of θ/L vs θ, the curve is cut off below 0.5. (f) Corresponding plot of L vs θ showing curve is cut off well below 0.5.

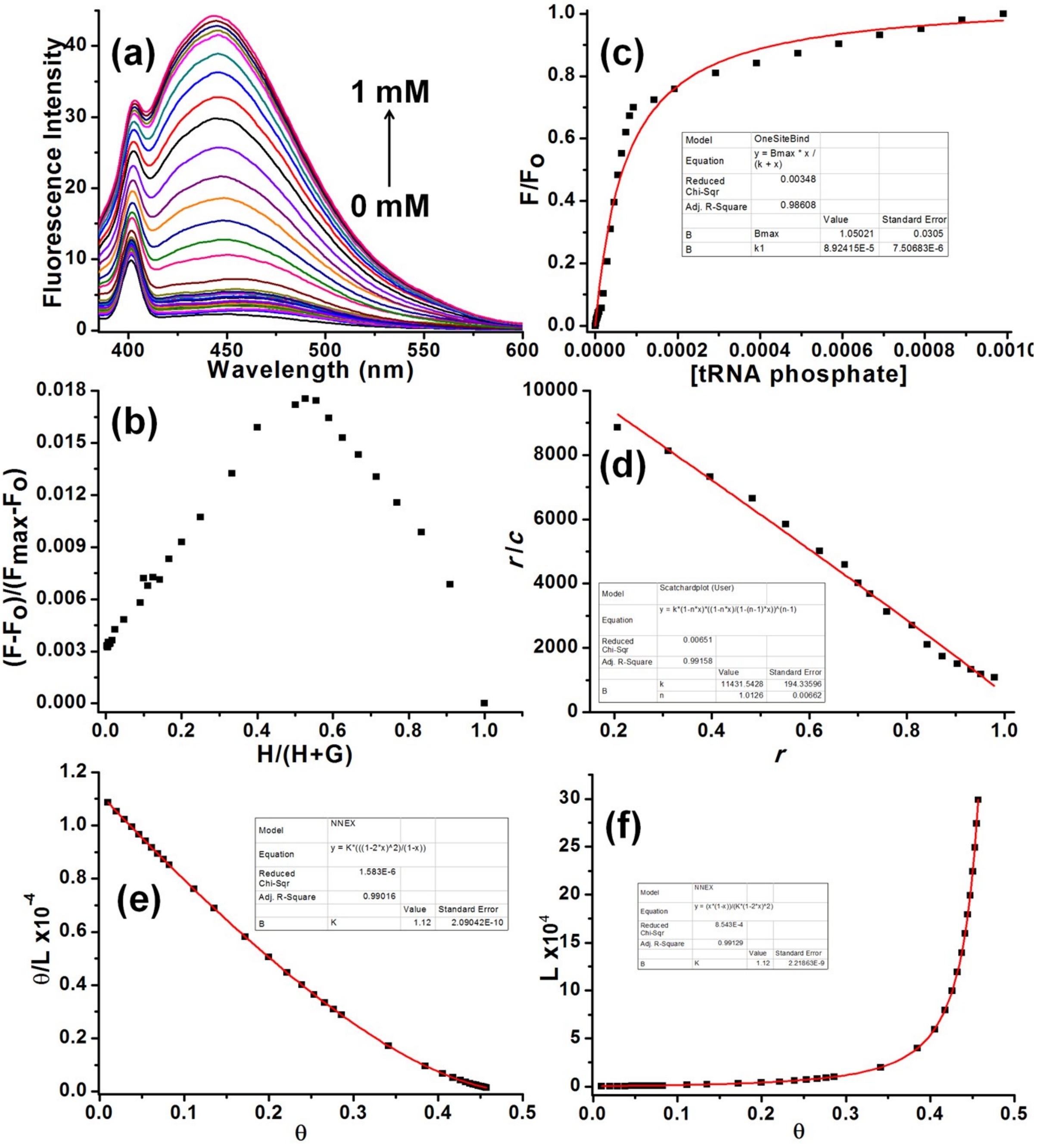

Figure S16. Fluorescence titration of probe **3** with tRNA from Baker's yeast. (a) Emission spectra (excitation at 350 nm) of Probe 3 (10 μM) upon addition of tRNA from Baker's yeast at pH 7.4 (0.01 M HEPES buffer, 25°C). (b) Assessment of the stoichometry of the RNA complex of **3** via Job plot analysis; [**3**] + [RNA] = 10 μM, pH 7.4 (10 mM HEPES buffer), 25ºC. (c) Corresponding binding isotherm of titration. (d) Scatchard plots, *r*/*c* vs *r*; *r* = ligand-to-RNA ratio, obtained from spectrofluorometric titrations of compound **3**-RNA in HEPES buffer (pH 7.4). The experimental data points were fitted to the model of McGhee and von Hippel. (e) Plot of θ/L vs θ, the curve is cut off below 0.5. (f) Corresponding plot of L vs θ showing curve is cut off well below 0.5.

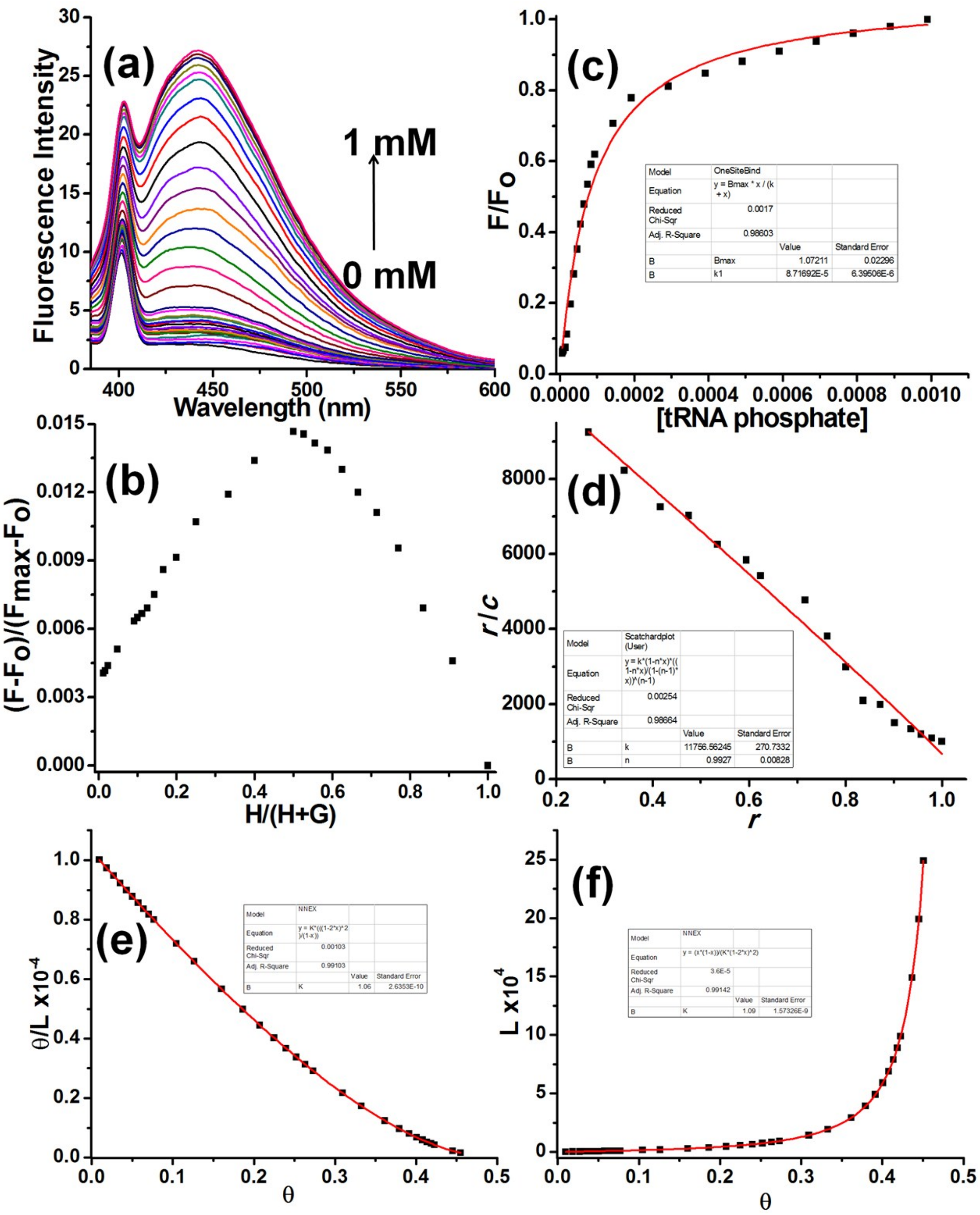

Figure S17. Fluorescence titration of probe **1** with tRNA from torula yeast. (a) Emission spectra (excitation at 350 nm) of Probe 1 (10 μM) upon addition of RNA from torula yeast at pH 7.4 (0.01 M HEPES buffer, 25ºC). (b) Assessment of the stoichometry of the RNA complex of **1** via Job plot analysis; [**1**] + [RNA] = 10 μM, pH 7.4 (10 mM HEPES buffer), 25ºC. (c) Corresponding binding isotherm of titration. (d) Scatchard plots, *r*/*c* vs *r*; *r* = ligand-to-RNA ratio, obtained from spectrofluorometric titrations of compound **1**-RNA in HEPES buffer (pH 7.4). The experimental data points were fitted to the model of McGhee and von Hippel. (e) Plot of θ/L vs θ, the curve is cut off below 0.5. (f) Corresponding plot of L vs θ showing curve is cut off well below 0.5.

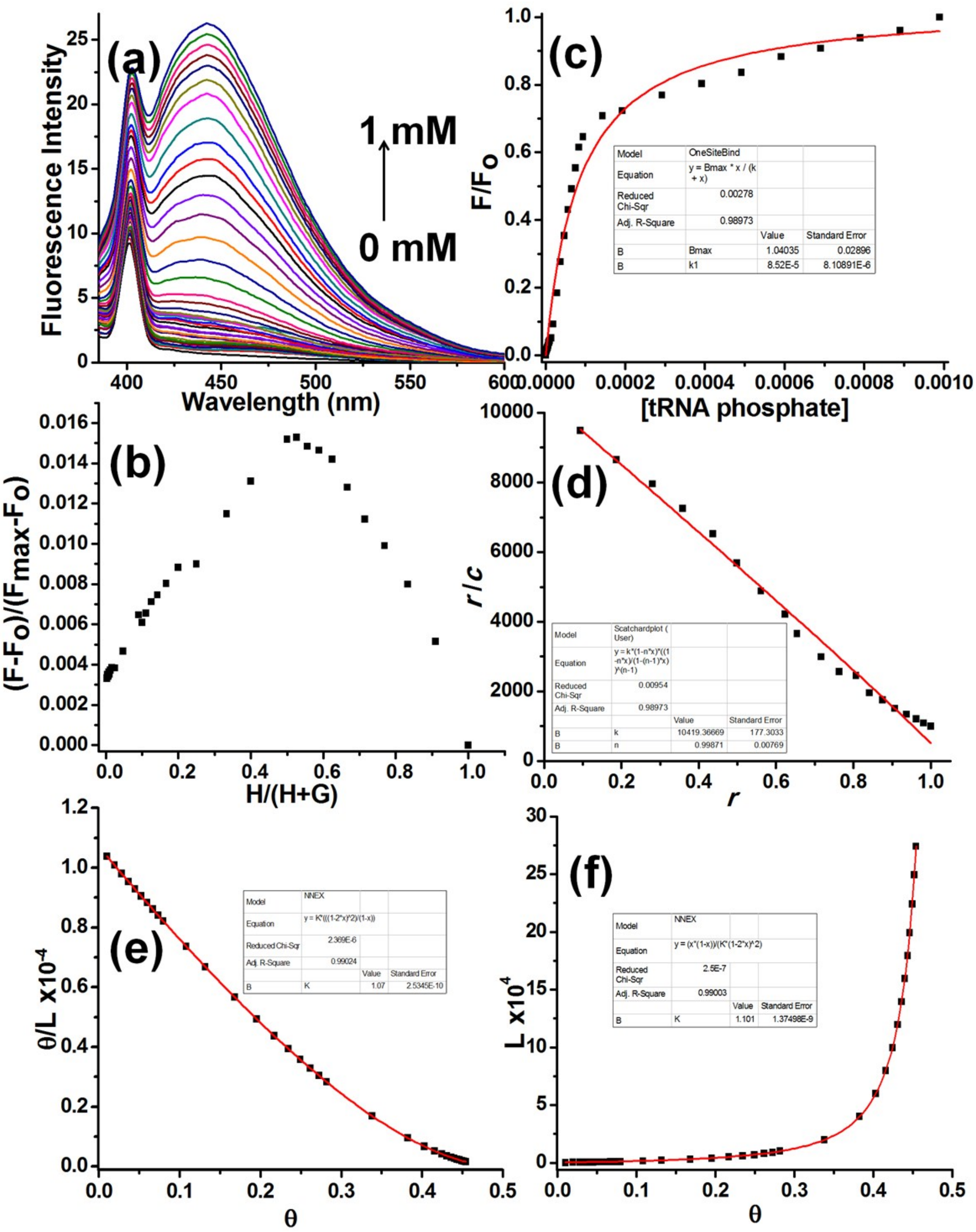

Figure S18. Fluorescence titration of probe **2** with tRNA from torula yeast. (a) Emission spectra (excitation at 350 nm) of Probe **2** (10 μM) upon addition of RNA from torula yeast at pH 7.4 (0.01 M HEPES buffer, 25°C). (b) Assessment of the stoichometry of the RNA complex of **2** via Job plot analysis; [**2**] + [RNA] = 10 μM, pH 7.4 (10 mM HEPES buffer), 25ºC. (c) Corresponding binding isotherm of titration. (d) Scatchard plots, *r*/*c* vs *r*; *r* = ligand-to-RNA ratio, obtained from spectrofluorometric titrations of compound **2-**RNA in HEPES buffer (pH 7.4). The experimental data points were fitted to the model of McGhee and von Hippel. (e) Plot of θ/L vs θ, the curve is cut off below 0.5. (f) Corresponding plot of L vs θ showing curve is cut off well below 0.5.

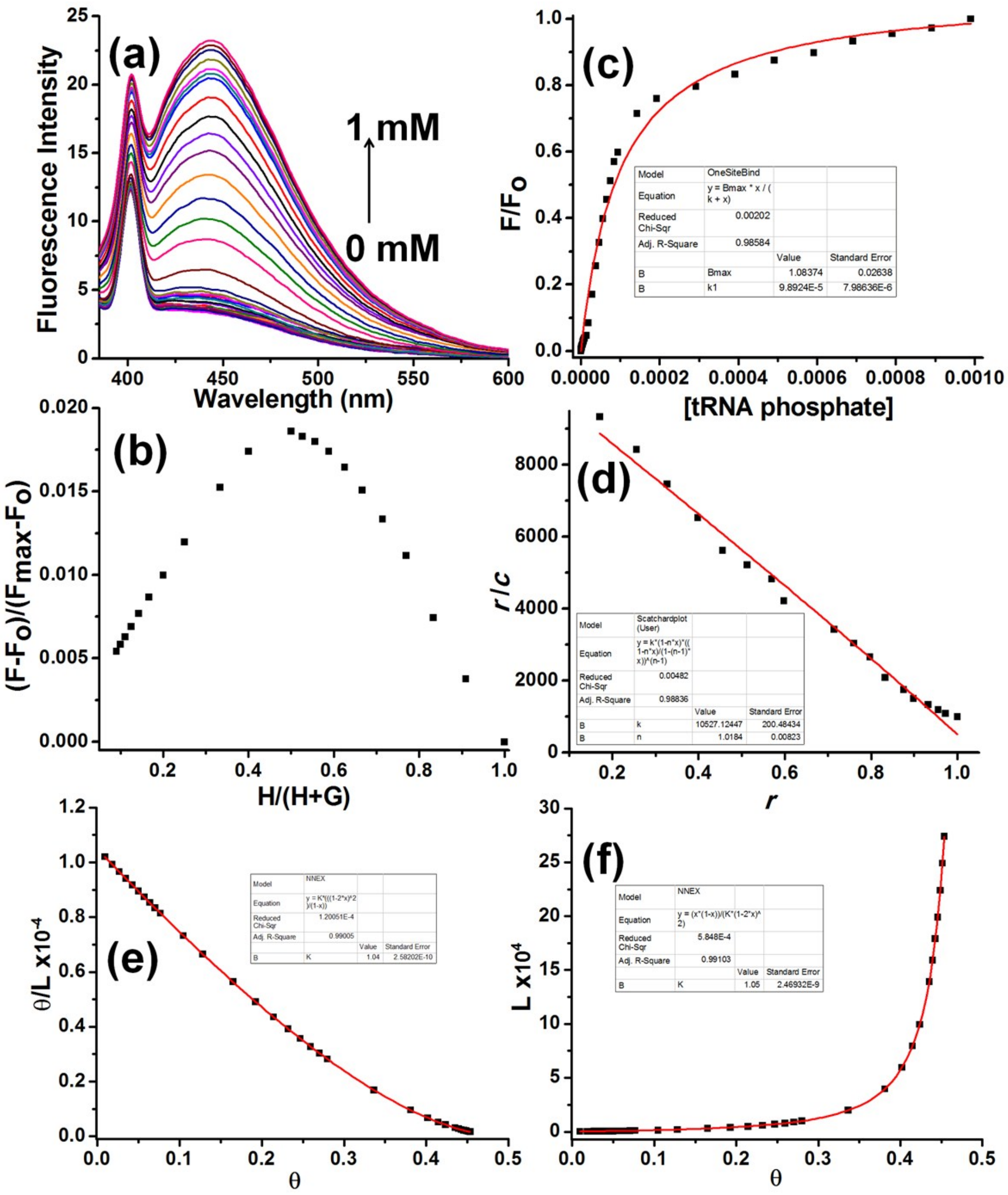

Figure S19. Fluorescence titration of probe **3** with tRNA from torula yeast. (a) Emission spectra (excitation at 350 nm) of Probe **3** (10 µM) upon addition of RNA from torula yeast at pH 7.4 (0.01 M HEPES buffer, 25°C). (b) Assessment of the stoichometry of the RNA complex of **3** via Job plot analysis; [**3**] + [RNA] = 10 µM, pH 7.4 (10 mM HEPES buffer), 25ºC. (c) Corresponding binding isotherm of titration. (d) Scatchard plots, *r*/*c* vs *r*; *r* = ligand-to-RNA ratio, obtained from spectrofluorometric titrations of compound **3**-RNA in HEPES buffer (pH 7.4). The experimental data points were fitted to the model of McGhee and von Hippel. (e) Plot of θ/L vs θ, the curve is cut off below 0.5. (f) Corresponding plot of L vs θ showing curve is cut off well below 0.5.

6. (TD-)DFT Calculation Results

The theoretical fluorescence spectrum has its meaning that all the intercalation structures are responsible for entire signal, not specific set of stacked bases, violating the neighbor-exculsion principle. Furthermore, the HOMOs and LUMOs shown in Figures S20 – S22 imply irrevalance of ribose-phosphate backbone in the excited states.

Even though the theoretically reproduced fluorescence (Figure 3) reasonably agrees with the experimental result, there are some reasons of small mismatch between them for ~20 nm. Firstly we assumed that all the structures show the same wavelength and oscillator strength when they have same stacking pairs, but they can actually show somewhat different values with different environments. For example, we optimized the 1st excited states of A-**1**-G and C-**1**-G with different structures obtained from different part of the same RNA. While the values (424.5 nm / 0.002) of A-**1**-G with the different structure are not quite different from the ones in Table S1, C-**1**-G with the different structure gives some deviations (426.2 nm / 0.005) from the corresponding values in Table S1. Another possibility would be attributed to other neighboring parts of the same RNA and probe molecules; ribose-phosphate backbone from other parts of the RNA nearby can interact with bases instead of water molecules. Probe molecules nearby also can surround the system, replacing water molecules. However, the latter two possibilities were not considered here due to cost-ineffectiveness from system size.

To verify the accuracy of the M06 results, we performed single point calculations using PBE0/6-31G* with the 1st excited state optimized structures based onthe M06/6-31G* because PBE0 is known to provide good description of charge-transfer excited states for the stacked bases.[6,7] Also, it is a good selection for excited state calculations for stacked aromatic molecules.[8]

The results in Table S1 and Figure S22 show that PBE0 gives similar oscillator strengths and excitation wavelengths with almost same HOMOs and LUMOs as those of M06 results. M06 has enough percentage of Hartree-Fock exchange (27%) to avoid spurious charge transfer (CT). Note that PBE0 has 25%. Thus, we can say that M06 describes CT excited states with similar extent of accuracy as PBE0. Besides, one more crucial point here is the description of π-π interaction for intercalation. With M06 functional we describe correctly the mid-range correlation energy, which is necessary to describe the π-π interactions.[9,10]

The oscillator strengths for vertical excitation to the 1st excited states and the corresponding wavelengths (0.005, 0.036, 0.042 and 0.023 / 311.5, 301.1, 295.2 and 315.2 nm for G-1-A, U-1-A, U-1-C and U-1-G, respectively) can be compared with the values of A-1-G, A-1-U, C-1-U and G-1-U (0.030, 0.020, 0.035, 0.047 / 320.1, 303.8, 297.7, 315.1 nm, respectively). The oscillator strengths which are generally known to be sensitive (while the significant change in magnitude is not so critical) are somewhat different between the opposite pairs, but the wavelengths are similar. Despite some

difference in oscillator strengths, the absorption spectrum of each fragment, which put all excitations from ground to nth excited states (n = 1, 2 and 3) together, is found to be very close to its counterpart.

Table S1. M06/6-31G* results for vertical excitations of diverse nucleobase stacking pairs from the optimized ground state and for energy release from the 1st excited state geometries.

	M06/6-31G*				PBE0/6-31G*	
**structure**	**absorption energy/nm**	**oscillator strength**	**fluorescence energy/nm**	**oscillator strength**	**fluorescence energy/nm**	**oscillator strength**
A-**1**-A	302.9	0.033	392.7	0.034		
A-**1**-C	307.3	0.050	386.7	0.055		
A-**1**-G	320.1	0.030	426.7	0.025		
G-**1**-A	311.5	0.005				
A-**1**-U	303.8	0.020	489.7	0.005	476.47	0.004
U-**1**-A	301.1	0.036				
C-**1**-G	301.4	0.028	443.6	0.003	446.96	0.001
C-**1**-U	297.7	0.035	494.9	0.012	485.86	0.007
U-**1**-C	295.2	0.042				
G-**1**-G	306.3	0.038	413.4	0.004	418.25	0.003
G-**1**-U	315.1	0.047	450.3	0.005	457.93	0.002
U-**1**-G	315.2	0.023				
U-**1**-U	314.9	0.013	502.5	0.005	488.28	0.004

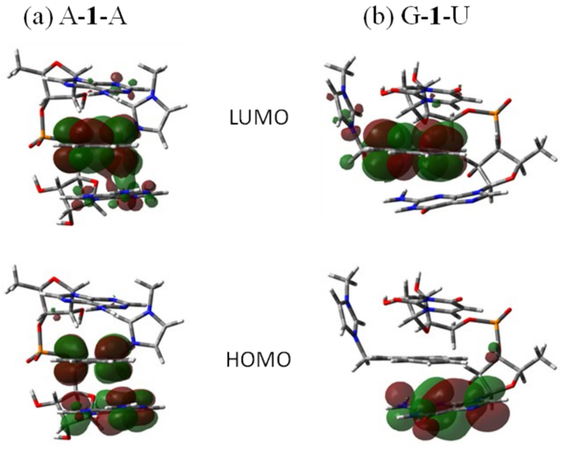

Figure S20. Representative HOMOs and LUMOs at the optimized 1st excited state geometry (a) A-**1**-A and (b) G-**1**-U.

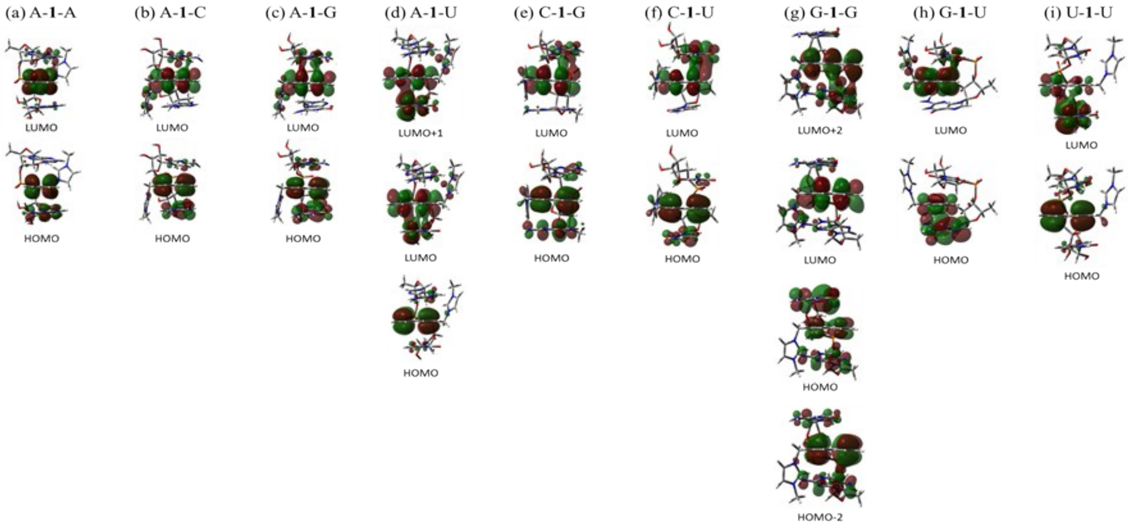

Figure S21. HOMOs and LUMOs for the optimized ground state structure of each stacking nucleobase pair with probe **1** intercalated.

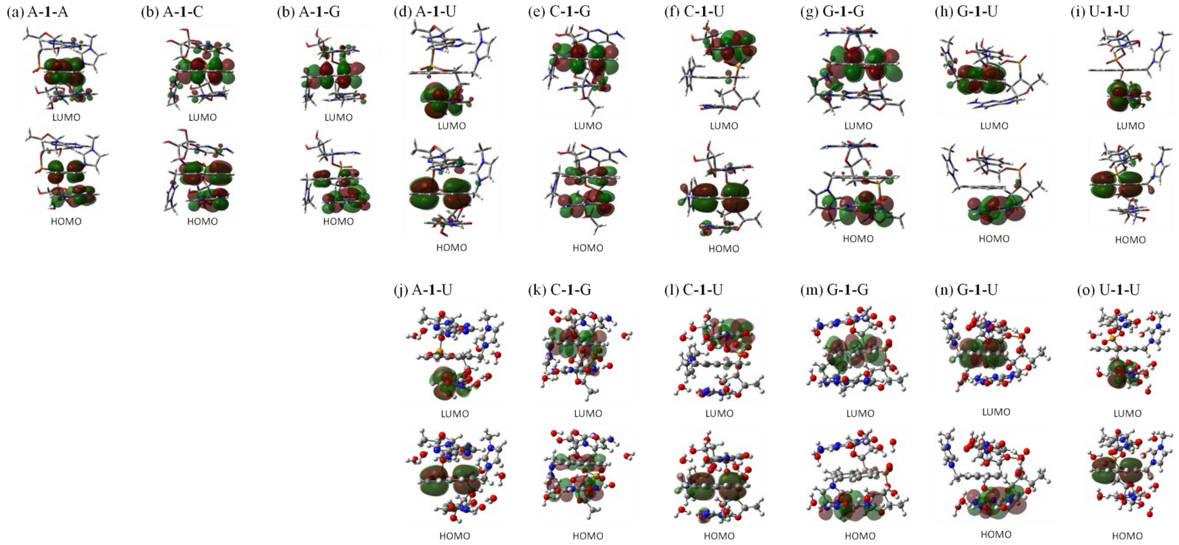

Figure S22. HOMOs and LUMOs for the optimized 1st excited state structure of each stacking nucleobase pair with probe **1** intercalated. (a) – (i) M06/6-31G* and (j) – (o) PBE0/6-31G* results, respectively

7. Circular Dichroism (CD) Studies

The CD spectra were collected at room temperature (25±0.2°C) using a Jasco made J-815 CD spectropolarimeter. Scans were from 350 to 200 nm with a resolution of 1 nm, with data sampling every 5 s. The 1 cm cell contained 1mM solution of the RNA (concentration of tRNA from baker's yeast and RNA from torula yeast was determined spectrometrically, ε_{260} = 9250 $M^{-1}cm^{-1}$, expressed as molarity of phosphate groups) in 0.01 M HEPES buffer (pH 7.4). 0.1 M solution of probe **1** was also prepared in 0.01 MHEPES buffer (pH 7.4). CD spectra were then recorded with pure tRNA and with addition of specific amount of probe **1**. The spectra were corrected for dilution, which was <10% at the end of the titration. CD spectra of RNA in the presence of various amount of probe **1** are shown in Figures S23-25. Binding constant determination K_a for RNA with Probe **1** was calculated.

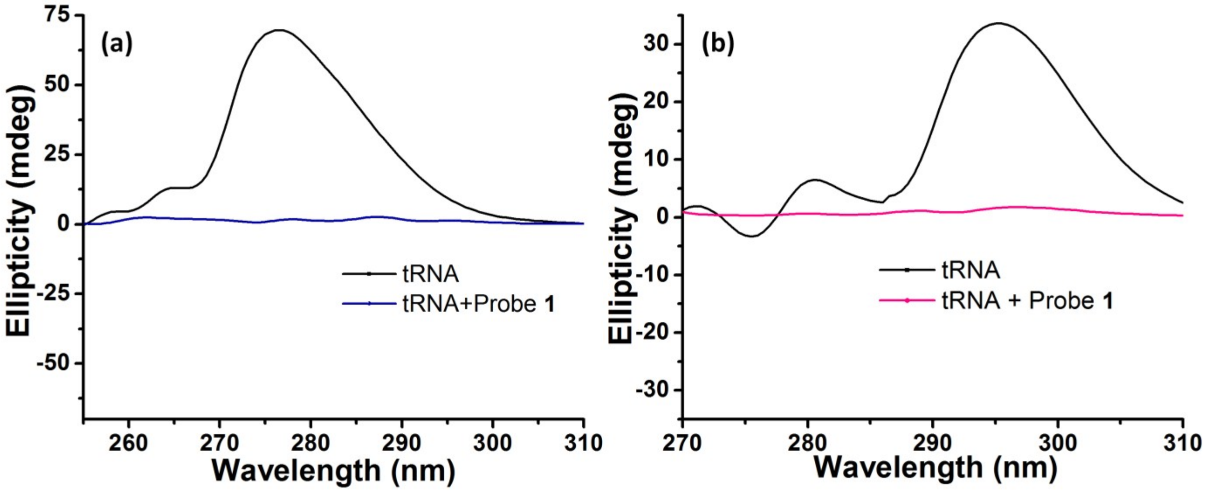

Figure S23. CD titration of probe **1** with RNA. (a) CD of tRNA from baker's yeast (2mM) with and without probe **1** (4.5 equiv.). (b) CD of RNA from torula yeast (2mM) with and without probe **1** (4.5 equiv.).

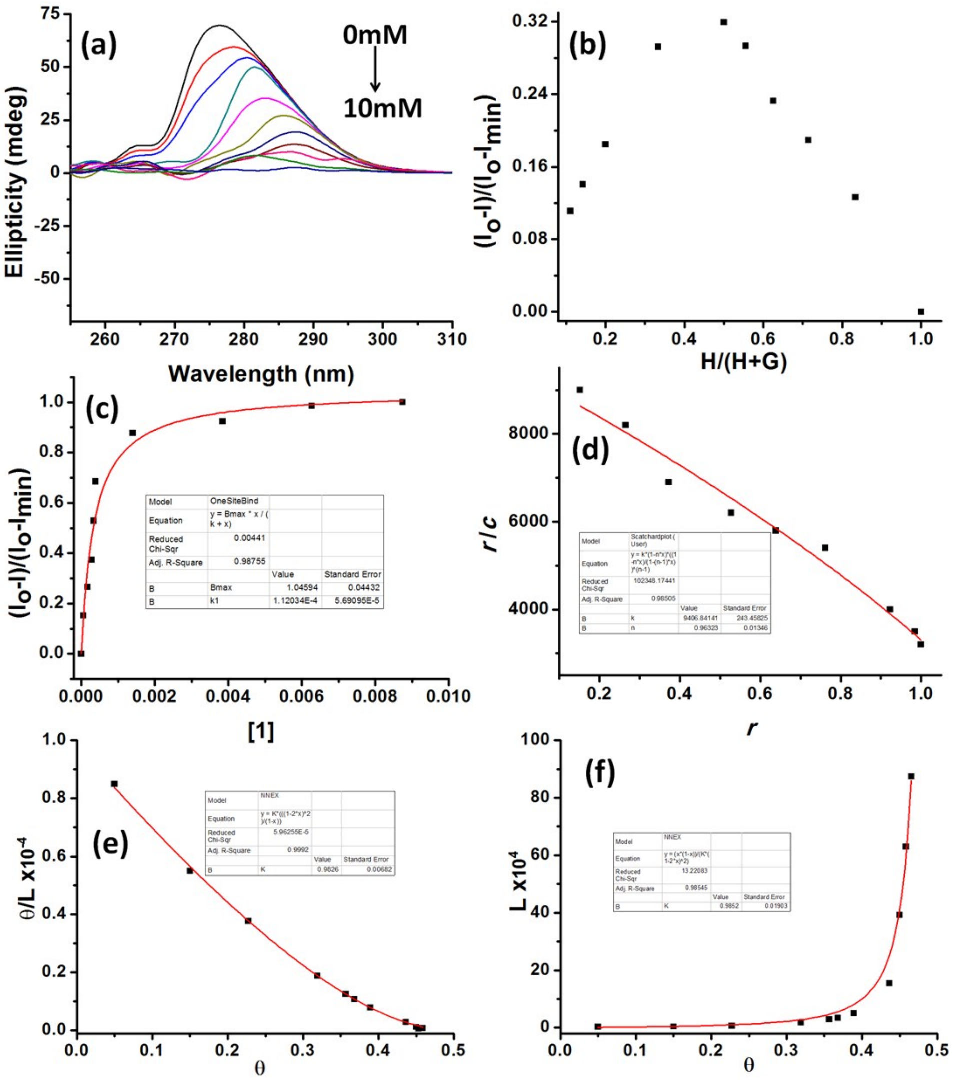

Figure S24. CD titration of tRNA from baker's yeast with probe **1**. (a) CD spectra of tRNA from Baker's yeast (2 mM) in the presence of and absence of Probe **1** at pH 7.4 (0.01 M HEPES buffer, 25°C). (b) Assessment of the stoichometry of the RNA complex of **1** via Job plot analysis; [**1**] + [RNA] = 1 mM, pH 7.4 (10 mM HEPES buffer), 25ºC. (c) Corresponding binding isotherm of titration. (d) Scatchard plots, *r*/*c* vs *r*; *r* = ligand-to-RNA ratio, obtained from spectrofluorometric titrations of compound **1**-RNA in HEPES buffer (pH 7.4). The experimental data points were fitted to the model of McGhee and von Hippel. (e) Plot of θ/L vs θ, the curve is cut off below 0.5. (f) Corresponding plot of L vs θ showing curve is cut off well below 0.5.

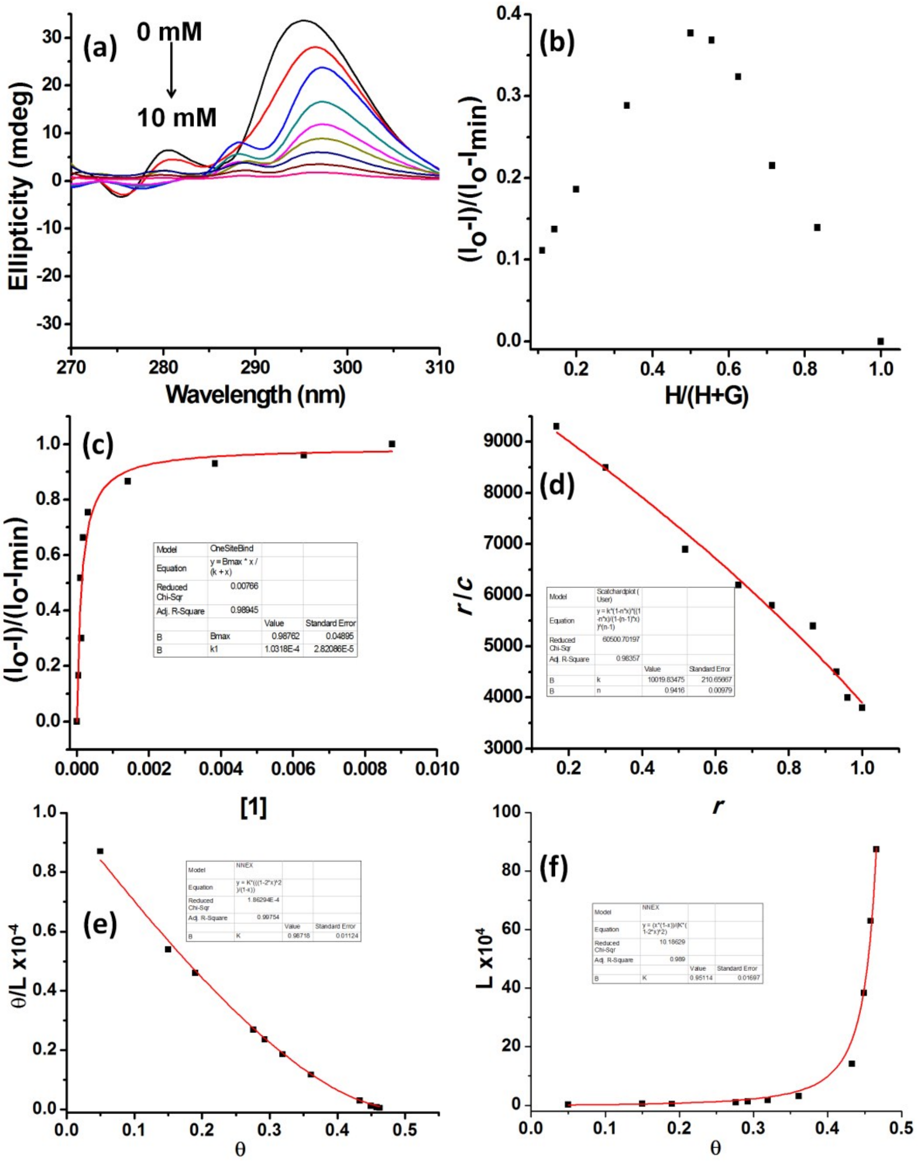

Figure S25. CD titration of RNA from torula yeast with probe **1**. (a) CD spectra of RNA from torula yeast (2 mM) in the presence of and absence of Probe **1** at pH 7.4 (0.01 M HEPES buffer, 25°C). (b) Assessment of the stoichometry of the RNA complex of **1** via Job plot analysis; [**1**] + [RNA] = 1 mM, pH 7.4 (10 mM HEPES buffer), 25ºC. (c) Corresponding binding isotherm of titration. (d) Scatchard plots, *r*/*c* vs *r*; *r* = ligand-to-RNA ratio, obtained from spectrofluorometric titrations of compound **1-**RNA in HEPES buffer (pH 7.4). The experimental data points were fitted to the model of McGhee and von Hippel. (e) Plot of θ/L vs θ, the curve is cut off below 0.5. (f) Corresponding plot of L vs θ showing curve is cut off well below 0.5.

8. ^{1}H NMR titration

Probe **1** was selected in order to monitor physical interaction through ^{1}H NMR experiment. tRNA (GCGCGCGCGC) and tRNA (AUAUAUAUAU) was used to investigate which base pair is responsible for interaction with the naphthalene moiety of probe **1**. ^{1}H NMR titration and 2D NOESY experiment was performed in 0.01M HEPES buffer pH 7.4 in D_2O. Addition of RNA to **1** (Figures S27-28) caused downfield shift of imidazolium protons $H_{b,c}$ (<0.3 ppm for H_b while <0.6 ppm for H_c) which might be due to electrostatic interaction with phosphate backbone of RNA. Downfield shift associated with splitting of naphthalene protons $H_{a,d,e}$ (<0.3 ppm) was observed, while upfield shifts of guanine and cytosine protons (<0.1 ppm) were noted for the RNA based on GCGCGCGCGC units (Figure S27). Similarly upfield shifts of adenine and uridine protons (<0.1 ppm) were observed for RNA with AUAUAUAUAU units when reacted with probe **1** (Figure S28).

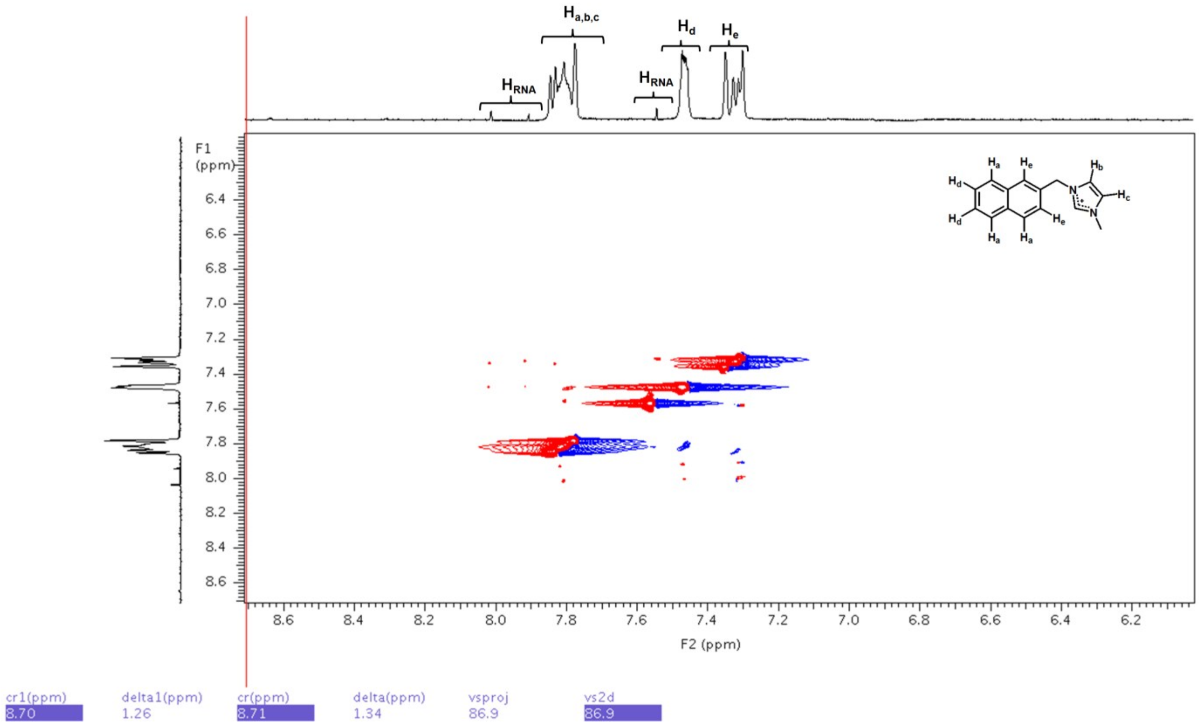

Figure S26. NOESY (600 MHz) spectra of Probe **1** + RNA from Baker's yeast. Probe **1** was dissolved in D_2O and solution of tRNA from Baker's yeast was added.

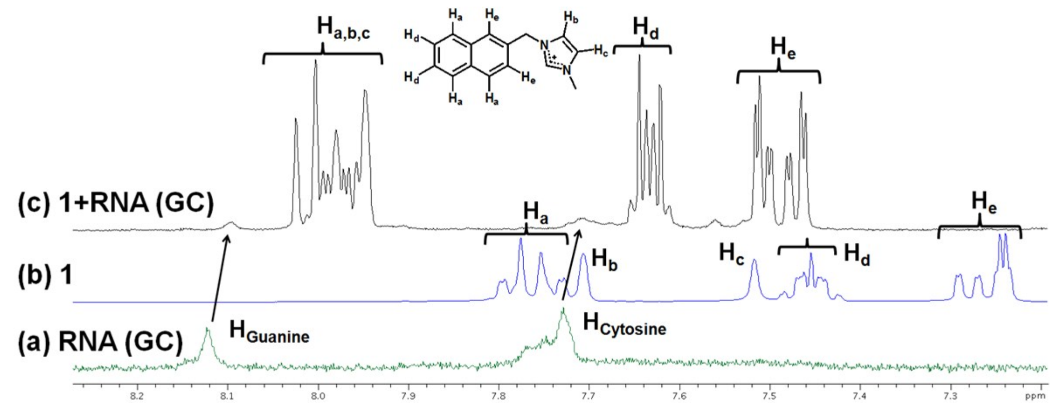

Figure S27. ^{1}H NMR titration. Partial 400 MHz ^{1}H NMR spectra for (a) GCGCGCGCGC, (b) Probe **1**, and (c) **1**+GCGCGCGCGC. Probe **1** was dissolved in D_2O and solution of GCGCGCGCGC was added**.**

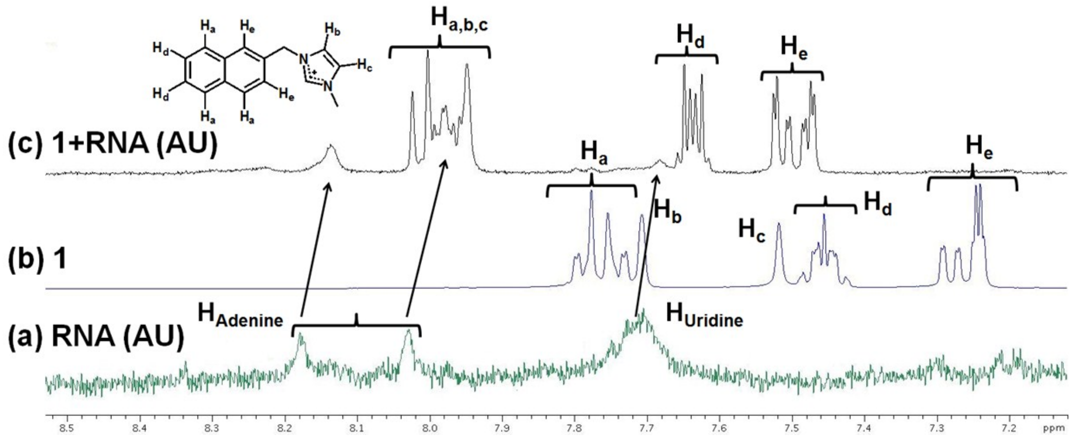

Figure S28. ^{1}H NMR titration. Partial 400 MHz ^{1}H NMR spectra for (a) AUAUAUAUAU, (b) Probe **1**, and (c) **1**+AUAUAUAUAU. Probe **1** was dissolved in D_2O and solution of AUAUAUAUAU was added.

9. MD Simulation Results

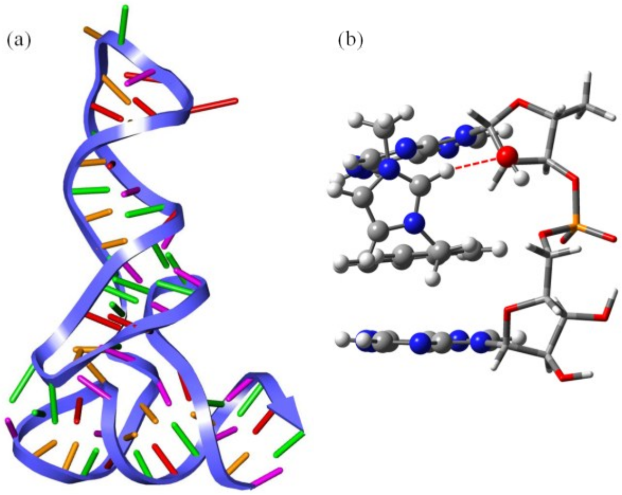

Figure S29. tRNA from baker's yeast. (a) Entire structure of tRNA of baker's yeast (red: adenine; violet: cytosine; green: guanine; orange: uracil; blue ribbon: ribose-phosphate backbone). (b) An intercalation structure considered for the (TD-)DFT study. Nucleobases, 2′-OH and probe **1** are represented as ball-and-stick model, while ribose-phosphate backbone is described as tube model. Water molecules are not shown here for clarity (white: H; blue: N; grey: C; red: O; orange: P; red-dotted line: hydrogen bond).

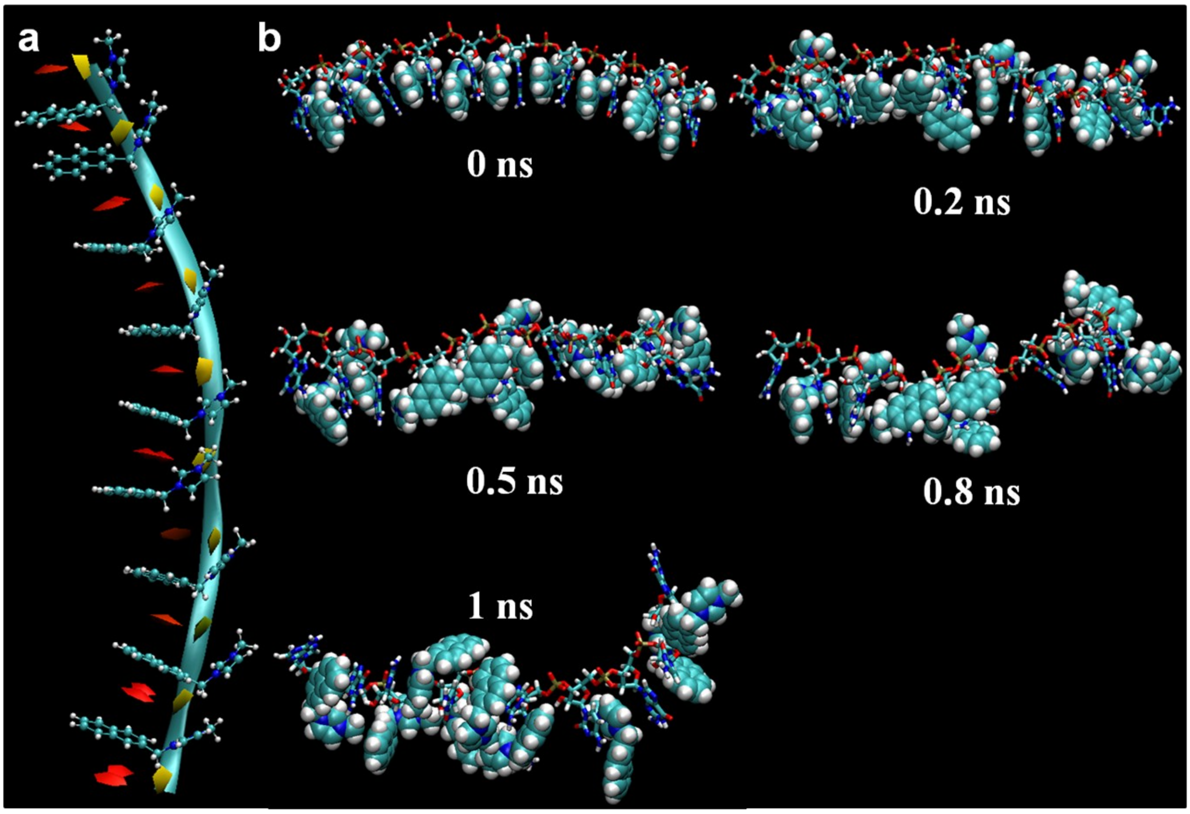

Figure S30. MD simulation Results (a) Schematic description of tRNA fragment of 10 nucleotides interacting with probe **1**: naphthalene moieties at intercalation sites and imidazoium moieties interacting only with 2′-OH of ribose (ribbon: phosphate backbone; ball-and-stick: probe **1**; yellow: ribose; red: nucleobase). (b) Snapshots of 1 ns MD simulation in NPT ensemble of the corresponding model (stick: RNA; vdW: probe **1**). Water molecules are removed for clarity.

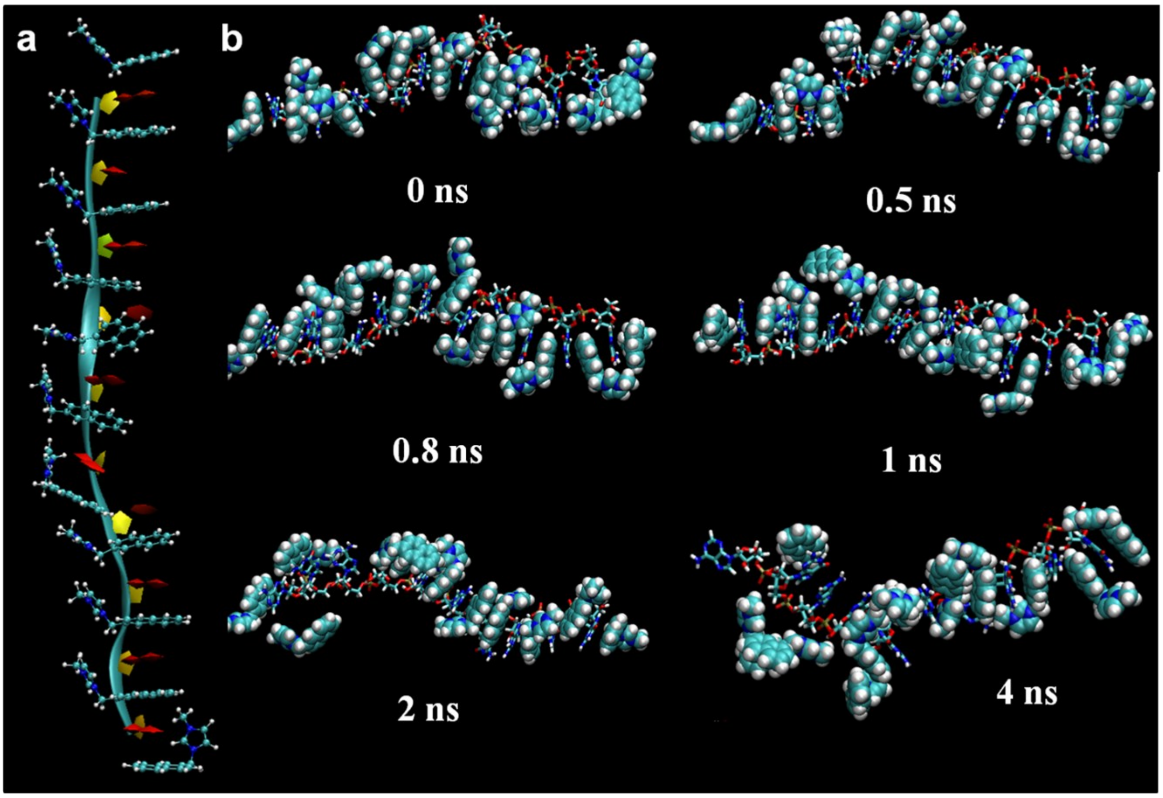

Figure S31. MD simulation Results (a) Schematic description of tRNA fragment of 10 nucleotides interacting with probe **1**: naphthalene moieties at intercalation sites and imidazoium moieties interacting only with phosphate (ribbon: phosphate backbone; ball-and-stick: probe **1**; yellow: ribose; red: nucleobase). (b) Snapshots of 4 ns MD simulation in NPT ensemble of the corresponding model (stick: RNA; vdW: probe **1**). Water molecules are removed for clarity.

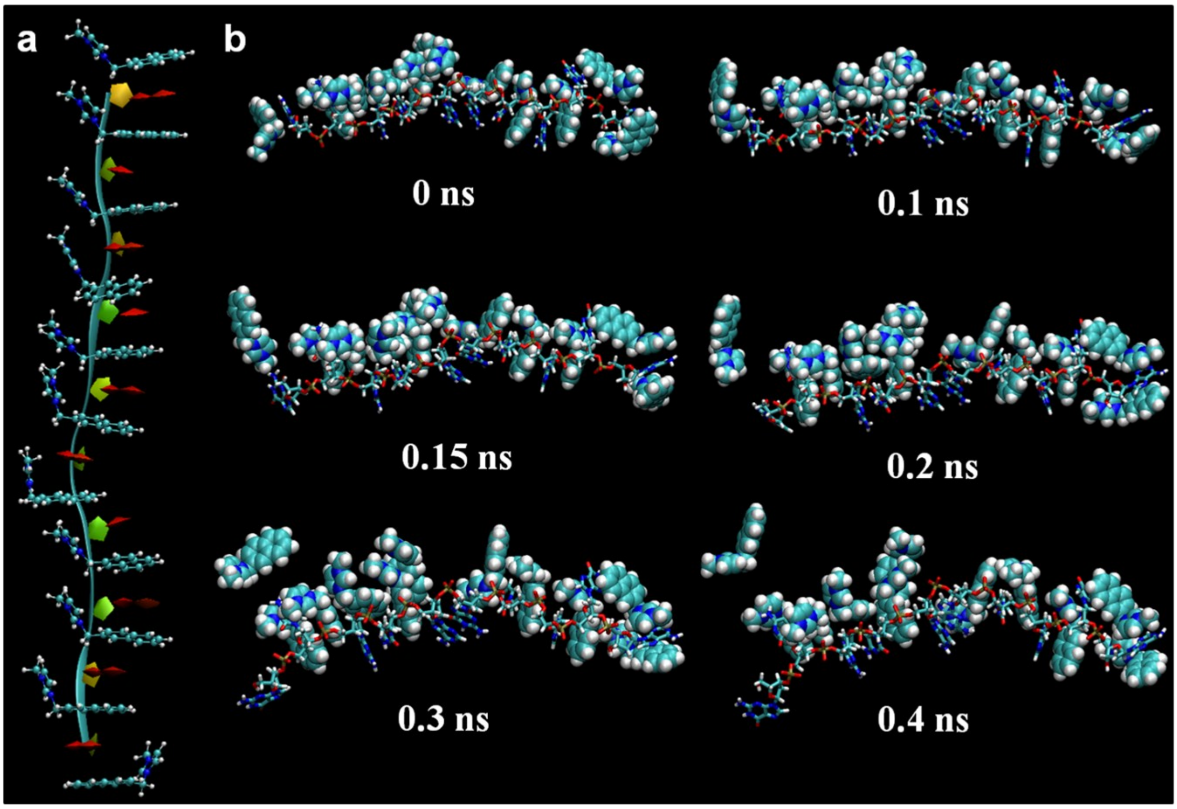

Figure S32. MD simulation Results (a) Schematic description of DNA fragment of 10 nucleotides interacting with probe **1**: naphthalene moieties at intercalation sites and imidazoium moieties interacting only with phosphate (ribbon: phosphate backbone; ball-and-stick: probe **1**; yellow: deoxyribose; red: nucleobase). (b) Snapshots of 0.4 ns MD simulation in NPT ensemble of the corresponding model (stick: DNA; vdW: probe **1**). Water molecules are removed for clarity.

The snapshots in Figure 5 is the MD simulation result in which three of eleven probe **1** molecules interact with oxygen atoms on phosphate backbone, and others interact with 2′-OH of ribose. The probe molecule only stacked on the end is solvated out within 1 ns. Then, two other probe molecules intercalated in between the nucleobases are solvated out further after 9 ns. After 10 ns, one stacked and 7 intercalated molecules stay in the initial positions. One can see that even after 15 ns, all the 7 molecules are still interacting with the RNA.

Totally different results are obtained in other two trajectories with different initial structures in which all the probe molecules interact only with either 2′-OH or phosphate oxygen (Figures S30 and S31). In the former case, all the probe molecules escape intercalation sites and are solvated out within 1 ns. The latter case shows that only two of nine intercalated probe molecules remain in the original positions after 4 ns simulations. The similar phenomenon is observed for DNA (Figure S32); almost all the intercalation structures are broken, only simply π stacking remaining, within 0.4 ns. It can be argued, therefore, that the intercalation structure is not able to stand strong in the cases of imidazolium moieties directing toward only one side, right (ribose) or left (phosphate); instead, the

intercalation structure can maintain long enough when both phosphate and ribose are interacting with probe molecules.

For further analasis, we investigated root-mean-square deviation (RMSD) of the distances between each intercalating probe molecule and the corresponding intercalated stacked bases for both RNA- and DNA-sensor systems. The DNA/RNA-sensor distance for each probe molecule was defined as the distance between center of intercalated bases and center of the naphthalene moiety. Here we depict the result of five ensembles of additional 10 ns simulations for the RNA-sensor system carried out from the point after the 5 ns equilibration simulation. As shown in Figure S33, the probe molecule remained intercalated between the adjacent RNA bases at the end of the simulation. Root-mean-square deviation (RMSD) of the separated distances between the probe molecule and stacked bases shows a steady behavior, showing the stability of sensor-intercalated geometry.

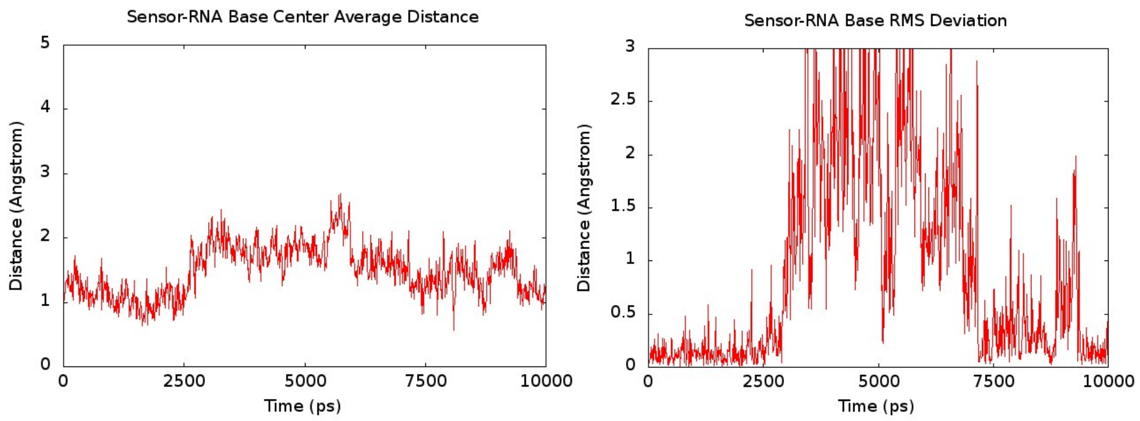

Figure S33. Averaged value and RMSD of the separated distances between the center of the intercalating naphthalene moiety and the center of the intercalated stacked bases of the RNA-sensor system from five different simultaneous 10 ns simulations carried out after 5 ns equilibriation of the sample. The sensor molecule has been partially detached from RNA base at 3.5 ns and intercalated again at 7 ns.

Seven simultaneous 1 ns simulations were performed with DNA-sensor system for comparison. Although the timescale is 10 times shorter than that of RNA, the DNA-sensor intercalated system is shown to be quite unstable, being detached after 0.5 ns (Figure S34).

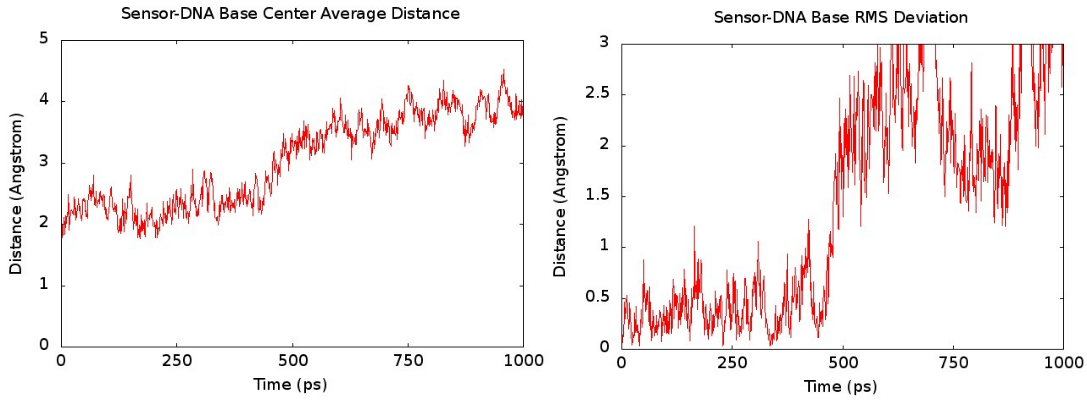

Figure S34. Average distance and RMSD data of the DNA-sensor system from seven simultaneous 1 ns simulations. Note that the molecule has been detached from the DNA strand after 0.5 ns.

Here we also show the result of 15 ns RNA-sensor system simulation. The distance between sensor and RNA was defined as the distance between the center of maphthalene moiety and the corresponding center of stacked bases. The average distance and RMSD for 15 ns simulations for the RNA-sensor system are given in Figure 35. Some sensors were detached from RNA, but soon found to be re-attached. Therefore, the exchange between intercalating and de-intercalating process could essentially be equilibrated. The root mean square fluctuation (RMSF) is also depicted in Figure S36. The drifting was also found to be minor.

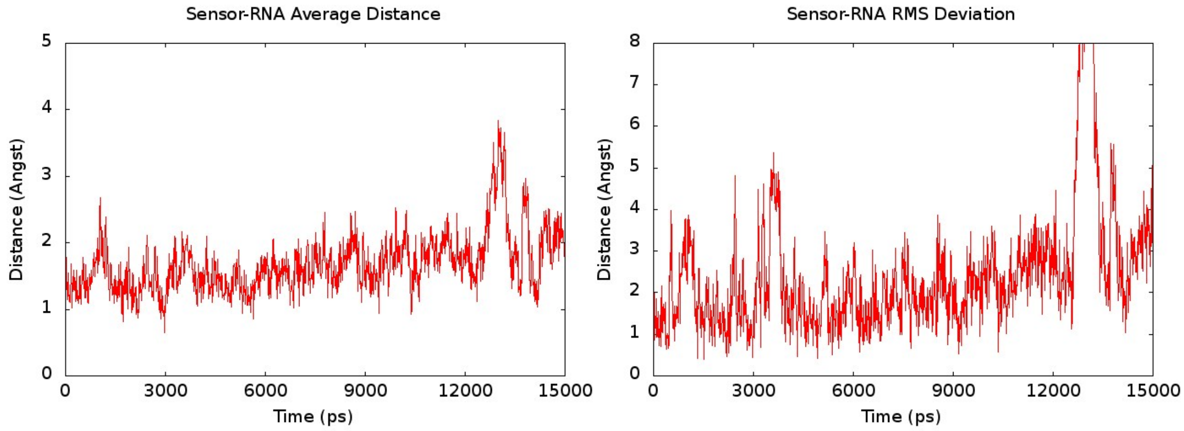

Figure S35. Average distance and RMSD for 15 ns simulations for the RNA-sensor system.

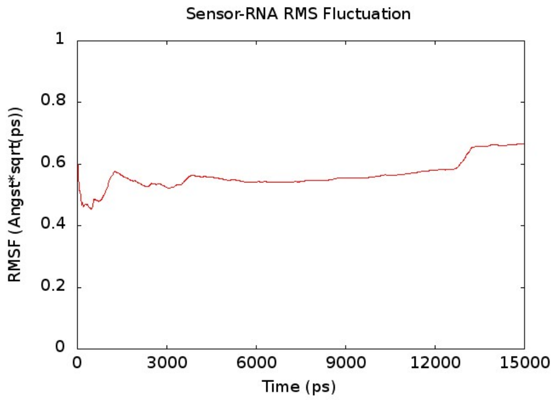

Figure S36. RMSF for the distances from the intercalating sensors (naphthalene moiety) to the centers of the corresponding intercalated bases for 15 ns simulations for the RNA-sensor system.

10. References

1. D. F. Eaton, *Pure & Appl. Chem.,* 1998, **60**, 1107-1114.
2. R. Mart, C. N. N'soukpoé-Kossi, D. M. Charbonneau, L. Kreplak, H-A. Tajmir-Riahi, *Nucl. Acids Res.,* 2009, **37**, 5197-5207.
3. (a) K. A. Connors, *Binding Constants*: *The Measurement of Molecular Complex Stability,* Wiley, New York, **1987**. (b) OriginLab 8.0, OriginLab Corporation, Northampton, MA, **2003**.
4. (a) J. D. McGhee, P. H. von Hippel, *J. Mol. Biol.* 1974, **86**, 469–489. (b) M. S. Rocha, *Biopolymers*, 2009, **93**, 1-7.
5. J. A. Schellman, H. A. Reese, *Biopolymers*, 1995, **39**, 161-171.
6. F. Santoro, V. Barone, R. Improta, *Proc. Natl. Acad. Sci. USA*, 2007, **104**, 9931-9936.
7. F. Plasser, H. Lischka, *Photochem. Photobiol. Sci.* 2013, **12**, 1440-1452.
8. M. Kolaski, C. R. Arunkumar, K. S. Kim, *J. Chem. Theory Comput*. 2013, **9**, 847-856.
9. Y. Zhao, D. G. Truhlar, *J. Chem. Theory. Comput.* 2007, **3**, 289-300.
10. Y. Zhao, D. G. Truhlar, *Theor. Chem. Acc.* 2008, **120**, 215-241.
